# Redox activation of excitatory pathways in auditory neurons as mechanism of age-related hearing loss

**DOI:** 10.1016/j.redox.2020.101434

**Published:** 2020-01-20

**Authors:** Francis Rousset, German Nacher-Soler, Marta Coelho, Sten Ilmjarv, Vivianne Beatrix Christina Kokje, Antoine Marteyn, Yves Cambet, Michael Perny, Marta Roccio, Vincent Jaquet, Pascal Senn, Karl Heinz Krause

**Affiliations:** a Hearing and Olfaction Research Laboratory, Department of Pathology and Immunology, Faculty of Medicine, University of Geneva, Switzerland; b Department of Pathology and Immunology, Faculty of Medicine, University of Geneva, Switzerland; c READS Unit, Faculty of Medicine, University of Geneva, Switzerland; d Department of Biomedical Research (DBMR), University of Bern, Switzerland; e Department of Otorhinolaryngology, Inselspital Bern, Switzerland; f Department of Clinical Neurosciences, Service of ORL & Head and Neck Surgery, University Hospital of Geneva, Switzerland

**Keywords:** NADPH oxidase, Age-related hearing loss, Presbycusis, Glutamatergic signaling, Auditory neurons, Excitotoxicity, NOX, NADPH oxidase, ROS, Reactive Oxygen Species, ARHL, Age-related Hearing Loss, CICR, Ca^2+^ induced Ca^2+^ release, OC, Organ of Corti, SV, Stria Vascularis, SGN, Spiral Ganglion Neurons

## Abstract

Age-related hearing (ARHL) loss affects a large part of the human population with a major impact on our aging societies. Yet, underlying mechanisms are not understood, and no validated therapy or prevention exists. NADPH oxidases (NOX), are important sources of reactive oxygen species (ROS) in the cochlea and might therefore be involved in the pathogenesis of ARHL. Here we investigate ARHL in a mouse model. Wild type mice showed early loss of hearing and cochlear integrity, while animals deficient in the NOX subunit p22^phox^ remained unaffected up to six months. Genes of the excitatory pathway were down-regulated in p22^phox^-deficient auditory neurons. Our results demonstrate that NOX activity leads to upregulation of genes of the excitatory pathway, to excitotoxic cochlear damage, and ultimately to ARHL. In the absence of functional NOXs, aging mice conserve hearing and cochlear morphology. Our study offers new insights into pathomechanisms and future therapeutic targets of ARHL.

## Highlights

• Mice devoid of NADPH oxidase (NOX) activity are protected from age-related hearing loss.
• Cochlear NOX expression shows a similar pattern in mouse and human.
• NOX3, the predominant NOX isoform in the cochlea, is mostly expressed in auditory neurons.
• NOX-deficient auditory neurons show decreased transcription of glutamatergic pathway and are protected from excitotoxicity.
• NOX-mediated gene regulation within auditory neurons contributes to age-related hearing loss.

## Graphical abstract

**Figure:**
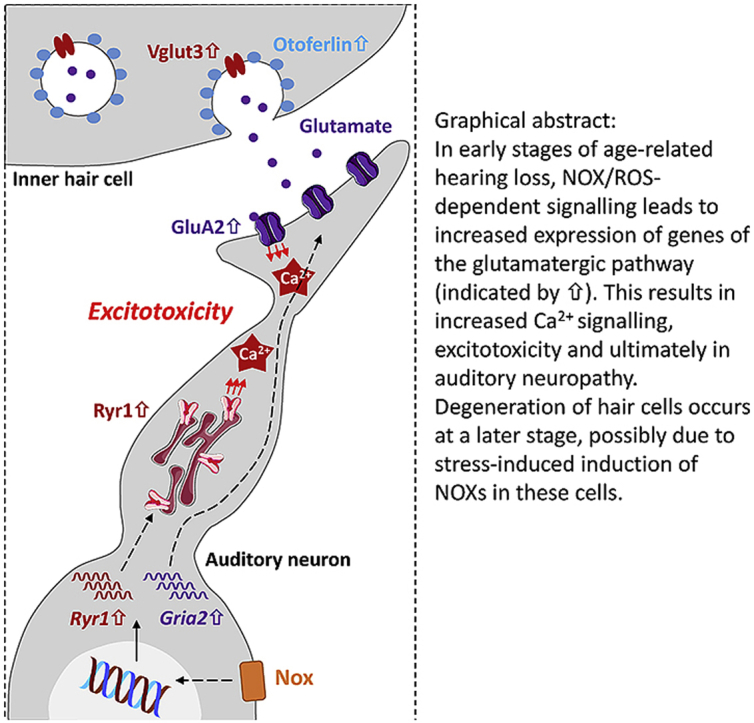

## 1 Introduction

Presbycusis or age-related hearing loss is a frequent, degenerative neurosensory disorder in ageing societies. Presbycusis affects more than a third of the elderly population at retirement age [1]. Hearing loss in this vulnerable population substantially contributes to cognitive decline, depression and social isolation [2,3] and represents an important socio-economic burden. Hearing aids and cochlear implants alleviate symptoms, but are not a causal treatment. Finding new treatments to reverse or slow down the progression of age-related hearing loss will have major consequences for the affected individuals and for society as a whole.

The etiology of presbycusis is multifactorial and involves a complex interaction of genes. It can be triggered by a variety of external and internal factors such as noise exposure, ototoxic molecules and medical conditions, among others [4]. The histopathological correlates of age-related hearing loss in the inner ear are the loss of mechanosensitive hair cells, spiral ganglion neurons, supporting cells and cells in the stria vascularis with a gradient from base to apex [5]. Therefore, the typical age-related hearing loss is predominantly affecting the higher frequencies, which are coded at the cochlear base.

Oxidative stress, i.e. high levels of reactive oxygen species (ROS), has traditionally been thought to lead to tissue damage and eventually cell death through non-specific protein, lipid and DNA oxidation. However more recent concepts rather favor a role of physiological ROS levels in cellular signaling [6], and a dysregulation of signaling through excessive ROS levels [7]. Abundant evidence demonstrates changes in the cochlear redox environment with age [8]. For instance, several antioxidant genes, including *SOD2*, *GST* or *UCP*, have been associated to age-related hearing loss [9] and genetic mouse models deficient for antioxidant genes, including *Nrf2* [10,11], *Sod1* [12] or genes regulating the mTOR pathway [13], show accelerated age-related hearing loss. Furthermore, age-related hearing loss (ARHL) has been shown to be slowed by supplementation with antioxidants in laboratory animals, and a few studies have investigated the effect of antioxidants supplements against ARHL in humans [14]. While a role of excessive oxidant generation has been widely described as causative of hearing loss, the exact sources of oxidants are unclear.

NADPH oxidases (NOX) are a family of enzymes whose main biochemical function is the production of ROS – namely superoxide radical anion O_2_^•-^ and H_2_O_2_. In mammals, the NOX family consists of seven isoforms (NOX1-5, DUOX1, 2). NOX have several subunits and the p22^phox^ subunit is crucial for the function of several NOX isoforms, namely NOX1 to NOX4. In fact, p22^phox^ is essential for NOX stabilisation and activity: the *CYBA* CRISPR knockout is devoid of ROS generation in NOX1, NOX2, NOX3 and NOX4 expressing cells [15]. Therefore, p22^phox^ is a master regulator of ROS generation as it regulates most NOX-derived oxidants. The biological function of NOX-derived ROS is broad, from host defense, to cellular signaling and hormone biosynthesis. NOX-derived ROS and in particular H_2_O_2_, resulting from superoxide dismutation are important second messengers in cell signaling. Through a reversible reaction with H_2_O_2_, cysteine residues are oxidized, changing the function of the respective protein [16]. Thereby, ROS regulates the activity of protein tyrosine phosphatases [17], permeability of ion channels [18] or affinity of transcription factors for their target DNA sequence [19] and thus regulate important physiological function in the cell (i.e. proliferation, differentiation, survival metabolism or motility). While NOX have important physiological functions in virtually all organ systems, an over-activation of these enzyme systems can lead to oxidative stress through overproduction of ROS and ultimately to oxidative stress-driven disease (e.g. fibrosis, cardiovascular disease, neurodegeneration). We hypothesize that in the inner ear, NOX activation results in hearing loss.

NOX3 is highly and exclusively expressed in the inner ear [20,21], and its function has been mainly attributed to otoconia formation in the developing vestibular system, whereas its function remains unknown in the cochlear tissues [20,22]. Nox3 mutant mice develop a vestibular deficiency, leading to a head-tilt phenotype. Mice with a loss of function mutation of the p22^phox^ subunit show a similar vestibular phenotype as NOX3 mutant mice with, in addition, a defect of innate immunity due to the absence of NOX2 [23]. Although growing evidence suggests a primary role of NOX and in particular NOX3 in different cochlear pathologies [24]; the contribution of NOX isoforms in age-related hearing loss has never been investigated. The fact that NOX3 is exclusively expressed in the inner ear makes it a prime target for interventions aiming at slowing down ROS production and consecutive cell-damage in the inner ear. While NOX3 is only found in the inner ear, more broadly expressed NOX family members are also present in the inner ear and may contribute to hearing and balance disorders. Indeed, NOX2 is highly expressed in microglia cells, which are abundant in spiral ganglia [25], and inhibition of microglia activation was protective in a mouse model of neomycin-induced hearing loss [26]. NOX4 is strongly expressed in vascular endothelium and could play a role in the stria vascularis. And indeed, NOX4 overexpressing transgenic mice show an increased sensitivity to noise-induced hearing loss [27].

Given the central role of p22^phox^ in NOX activity, p22^phox^ deficient mice are a particularly useful tool for studying the role of NOX in inner ear [23]. Indeed, considering the absence of Nox5 in the mouse, p22^phox^ deficient mice can be defined as pan-Nox-deficient mouse model [28]. The nmf333 mouse model (A.B6 Tyr + nmf333 – jax; A/J genetic background) harbors a missense mutation in *CYBA* leading to a functional inactivation of NOX1-4 [29]. The A/J strain shows a very early age-related hearing loss and is therefore an interesting model to study oxidative-related pathologies of the inner ear and to evaluate the effects of potential otoprotective drugs [30].

In the present study, the A/J mouse model (nmf333) was used to investigate the impact of NOX in the progression of age-related hearing loss. Our data demonstrate that p22^phox^ is a key regulator of age-dependent hearing loss. Mechanistically, we show that p22^phox^ regulates the calcium release from intracellular stores in auditory neurons and that deletion of p22^phox^ protects from excitotoxic insult in auditory neurons. Since, p22^phox^ is a key regulator of redox pathways due to its main function as controlling NOX activity, our data suggest a role of oxidant-derived NOX in age-related auditory neuropathy and show promises for novel therapeutic approaches for future treatment of age-related hearing pathologies.

## 2 Results

**Fig. 1:**
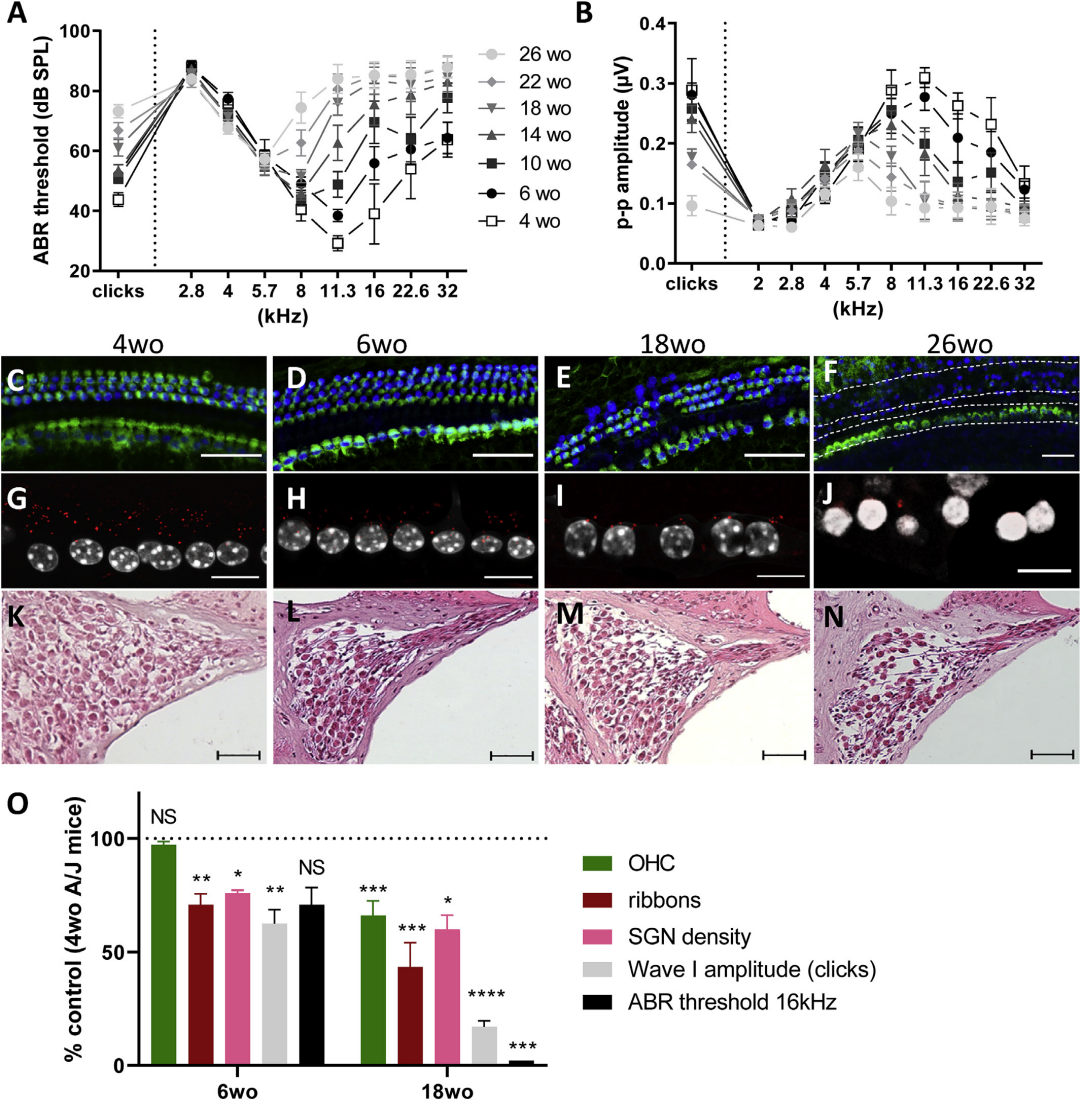
**Age-related hearing loss in wild type A/J mice: functional and morphological alterations in auditory neurons precede the loss of sensory epithelium.** A) Auditory brainstem response hearing thresholds of A/J mice were assessed from 4 to 26 weeks old animals, on a group of 11 WT A/J mice following clicks and pure tone frequencies stimulation. B) Elevation of hearing threshold is accompanied with a decrease of the ABR maximal amplitude (peak to peak amplitude, (p–p)), assessed at 75 dB SPL. C–F) Basal turn representative sensory epithelium immunostainings (Myo7a in green) of the WT A/J cochlea at 4 weeks old (C), 6 weeks old (D), 18 weeks old (E) and 26 weeks old (F). G-J) On the same samples, the number of synaptic ribbons between inner hair cells (nucleus in white) and spiral ganglion neurons was also determined using CtBP2 immunostaining (red dots), using 4 weeks old (G), 6 weeks old (H), 18 weeks old (I) and 26 weeks old (J) mice. K–N) Representative mid-modiolar hematoxilin-eosin staining of A/J mouse basal cochlear turns at 4 weeks old (K), 6 weeks old (L), 18 weeks old (M) and 26 weeks old (N). O) Bar graph showing the quantification of outer hair cells (green), synaptic ribbons (red), SGN density (pink), ABR wave I amplitude (light grey) and ABR threshold (black) in the 6 and 18 weeks old mice basal turn, relatively to the 4 weeks old group that serve as reference (100%). n = 11 animals. Scale bar (C–F) = 25 μm, (G–J) = 10 μm and (K–N) = 50 μm. (For interpretation of the references to color in this figure legend, the reader is referred to the Web version of this article.)

**A/J mouse as model for age-related hearing loss.** In order to characterize the early onset hearing loss in A/J mice, we investigated hearing thresholds starting over an age range from 4 to 26 weeks (Fig. 1A and B). Click-evoked auditory brain stem response (ABR) hearing thresholds were close to 45 dB SPL in young adult mice (4 weeks old) and progressed up to 75 dB SPL in old mice (Fig. 1A). The pure tone frequencies measurement (2–32 kHz) was more informative: while young mice audiogram was comprised between 2 and 32 kHz (with a threshold of 30 dB SPL at 11.3 kHz) (Fig. 1A; empty squares) we observed a progressive loss of hearing at high frequencies narrowing the hearing spectrum (from 32  kHz at 4 weeks to 5.7  kHz at 26 weeks old). Accordingly, maximal amplitude of the ABR decreased with age (Fig. 1B). The most important hearing loss was observed at around 11.3–16 kHz (Fig. 1A). Consistent with the high frequency hearing loss phenotype, we observed a progressive degeneration of the sensory epithelium from the base to the apical turn (Supplementary Fig. 1). While the apical turn was relatively well conserved in old mice, there was an important cellular degeneration in the basal region (Fig. 1C–F). This was accompanied by a dramatic decrease in the number of synaptic ribbons per inner hair cell (IHC) (Fig. 1G–J) and a marked decrease in the neuronal density in the spiral ganglion with age (Fig. 1K-N). Interestingly, the kinetics of degeneration, as observed in the basal turn of the cochlea, showed synaptic and post synaptic morphological alteration (decrease in number of ribbons per IHC, auditory neuron density and in the ABR wave I) occurring prior to the sensory epithelium degeneration (Fig. 1O; the dotted line represent control values obtained with mice at 4 weeks of age). Therefore, we conclude that A/J mice have an early onset age-related hearing loss which is mainly caused by neural degeneration and subsequent loss of the sensory epithelium.

**Fig. 2:**
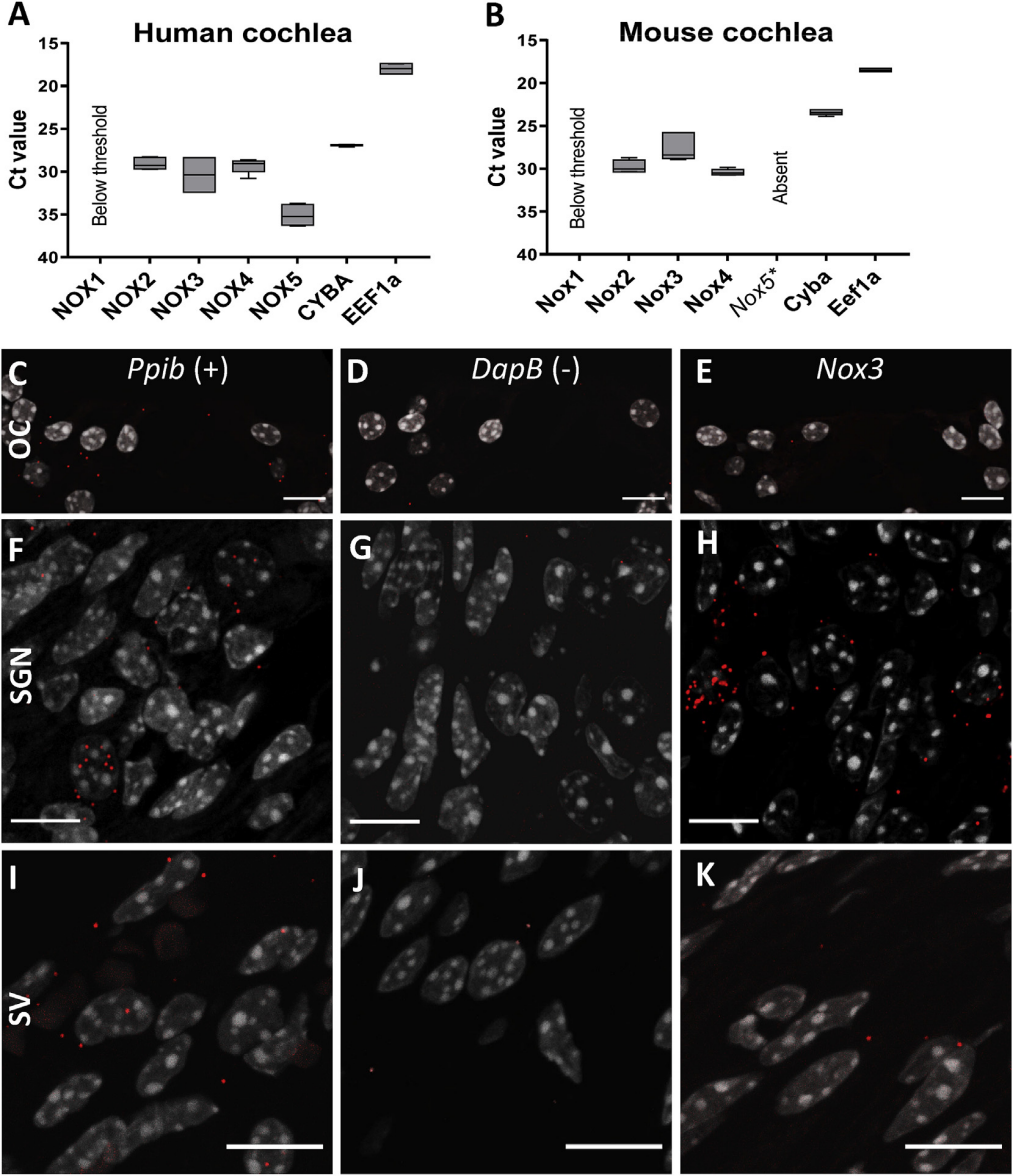
**Pattern of NOX mRNA expression in the mouse and human cochlea.** Expression level of Nox isoforms assessed by qPCR in human fetal cochlea (A) and 6 weeks old A/J mouse cochlea (B). Each graph shows the Ct values as observed for *NOX1* to *NOX5* and *CYBA* (p22^phox^). To note the absence of *Nox5* in mice. Level of expression of *NOX1* and *DUOX1/2* (not shown) was below the detection threshold. *EEF1* was used as housekeeping gene. n = 3 to 6 cochlea/group. (C–K) mRNA expression level of *Nox3* in the organ of Corti (OC) (E), the spiral ganglia (SGN) (H) and the stria vascularis (SV) (K) of a 6 weeks old WT A/J mouse as assessed by in situ hybridization (RNAscope®) (red dots). Peptidylprolyl isomerase B gene (*Ppib*) was used as positive control (C, F, I) and dihydrodipicolinate reductase (*DapB*) expressed in the bacteria E. Coli was used as negative control (D, G, J). Nuclei were stained with DAPI (grey). Pictures are representative from 3 independent experiments. (For interpretation of the references to color in this figure legend, the reader is referred to the Web version of this article.)

**Pattern of NOX expression in the cochlea.** While NOX3 is known to be highly enriched in the inner ear, the presence of other NOX isoforms was not studied [24,31]. Therefore, we addressed the expression of NOX isoforms in the cochlea of the human fetus (Fig. 2A) and the adult A/J mouse (Fig. 2B–K). By qPCR, we found relatively high levels of *NOX2*, *NOX3* and *NOX4* mRNA (CT values below 30) in both human (Fig. 2A) and mouse cochlea (Fig. 2B). In both species, *NOX1* and *DUOX1/2* levels in the cochlea were below detection threshold. *NOX5* levels in the human cochlea were low (Ct values 33–35). Note that *NOX5* gene is absent in mice and rats [28]. The fetal cochlear tissues used for real time qPCR is heterogenous and contained spiral ganglion neurons, as well as parts of the stria vascularis. We therefore aimed to obtain more precise information about the localization of the *NOX* mRNA. There are major issues with antibodies for the different NOX isoforms [32]. We therefore rather performed in situ hybridization (using the RNAscope technology) in order to localize the mRNA of the different isoforms (Fig. 2C–K). We compared expression of *Nox2*, *Nox3* and *Nox4* in three different subparts of the cochlea, namely the organ of Corti, the stria vascularis and the spiral ganglia. Signals with the *Nox2* and *Nox4* probes (Supplementary Fig. 2) were detected throughout the cochlea without a strong signal in specific parts. In contrast, the NOX3 probe gave a very strong signal in the spiral ganglia (Fig. 2H), comparable or even stronger than the control gene (*Ppib*, Fig. 2F; Supplementary Fig. 3). The *Nox3* signal also appeared above background in the stria vascularis (Fig. 2K), but not in hair cells (Fig. 2E).

**Fig. 3:**
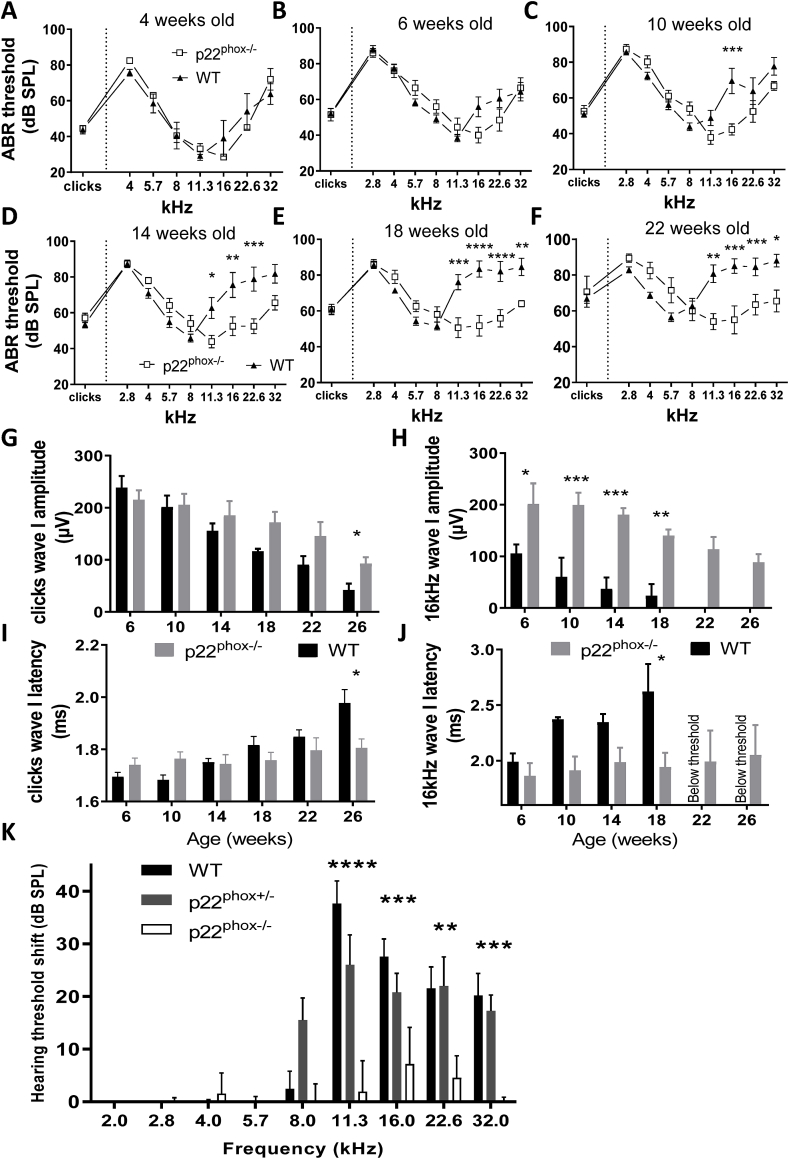
**p22**^**phox**^**-deficient mice are protected from early onset hearing loss.** A-F) Audiogram of A/J WT (black triangles) versus p22^phox^ deficient littermates (white squares) as determined by auditory brainstem response following clicks and pure tone frequencies stimulation, from 4 to 22 weeks old mice. A) 4 weeks old, B) 6 weeks old, C) 10 weeks old, D) 14 weeks old, E) 18 weeks old and F) 22 weeks old. G-H) Bar graph showing ABR wave I amplitudes of WT and p22^phox^−/− littermates at the indicated ages following G) click or H) 16 kHz pure tone stimuli at 75 dB SPL. I-J) Bar graph showing ABR wave I latencies of WT and p22^phox^ −/− littermates at the indicated ages following I) click or J) 16 kHz pure tone stimuli at 75 dB SPL. Note that at 16 kHz, the sound stimulus was below hearing thresholds of 22 and 26 weeks old WT (Below threshold). K) Bar graph showing hearing threshold shifts at 18 weeks old. n = 11 WT; 12 p22^phox^+/-; 8 p22^phox^−/−.

**p22**^**phox**^ **deficient A/J mice are protected from early onset hearing loss**. As several NOX isoforms are present in the inner ear, we next investigated hearing of A/J mice, harboring a loss of function mutation on the NOX1-4 subunit p22^phox^ (nmf333) (Fig. 3). For comparison, the results of wild type mice, as already described in Fig. 1, are added. Hearing thresholds of mice were investigated monthly from 4 to 22 weeks old (Fig. 3A–F). Four weeks old p22^phox^ mutant mice, exhibited an audiogram profile similar to wild type littermates, indicating that there is no basal difference in hearing between p22^phox^ mutant and WT mice (Fig. 3A). As already described in Fig. 1, from an age of 6 weeks, high frequencies hearing loss was observed in WT mice (Fig. 3B–F, Fig. 3K). In contrast, only minor hearing impairment was observed in mutant mice. In particular the high frequency hearing loss was almost completely prevented in p22^phox^ deficient animals up to 18 weeks old (Fig. 3K). In fact, the pattern of hearing loss was distinct in p22^phox^ deficient mice. At 22 weeks old, p22^phox^ mutant showed only a minor (statistically not significant) hearing loss. This minor hearing loss appeared to be equally distributed along the tonotopic axis (Fig. 2F). This is in contrast to the WT animal where the amplitude of the ABR recorded at 75 dB SPL consistently showed age-dependent decrease at high frequencies (Fig. 3H; Supplementary Fig. 4) and following click stimuli (Fig. 3G). In addition to the hearing threshold elevation, WT mice exhibited significant delay in the ABR wave I with age (Fig. 3I and J). Together, these experiments clearly demonstrate the role of p22^phox^-dependent NOX in age-related hearing loss in A/J mice.

**Fig. 4:**
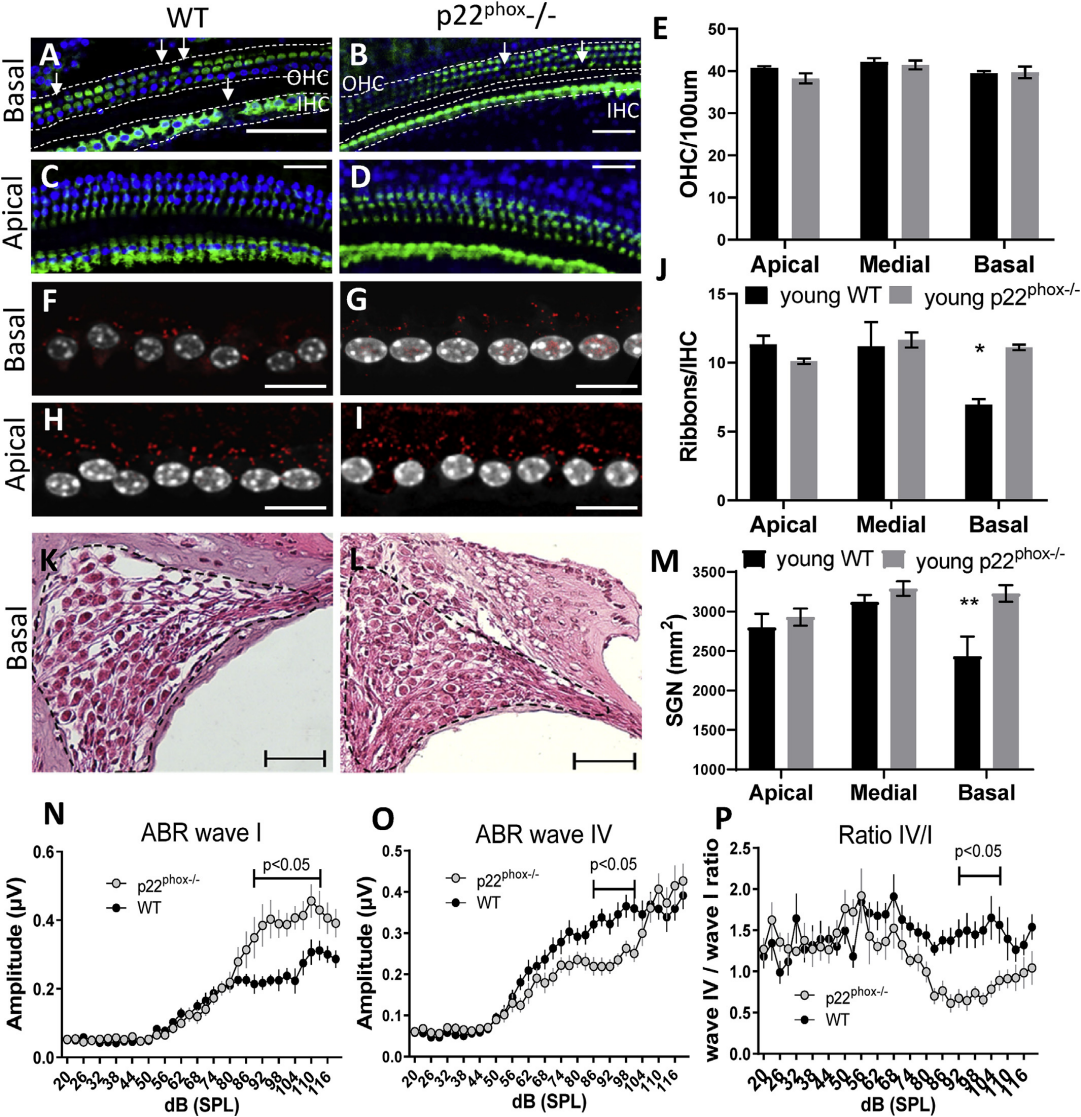
**p22**^**phox**^**deficiency prevents age-related auditory neuropathy.** A-D) Representative sensory epithelium immunostainings (Myo7a in green) of the basal (A–B) and apical turn (C–D) of the A/J WT (A and C) and p22^phox^ deficient (p22^phox^−/−) (B and D) cochleae, at the age of 6 weeks old. E) Bar graph showing the number of outer hair cells (OHC)/section of 100 μm. F–I) On the same samples, the number of synaptic ribbons between inner hair cells and spiral ganglion neurons was determined using CtBP2 immunostainings (red): F) and H) show respectively the WT mice basal and apical part of the cochlea. G) and I) show respectively the p22^phox^ deficient mice basal and apical turns of the cochlea. J) Bar graph showing the number of ribbons/inner hair cell (IHC). K-L) Representative mid-modiolar hematoxylin and eosin staining showing the medial turn of 6 weeks old WT (K) and p22^phox^ −/− littermate (L). M) Bar graph showing the density of spiral ganglion neurons (SGN)/mm^2^ in the different parts of the cochlea of WT and p22^phox^ −/− animals. n = 8 WT; n = 7 p22^phox^ −/−. N–O) ABR wave I (N) and ABR wave IV (O) amplitudes generated following clicks stimulation from 20 to 120 dB SPL (3 dB SPL increments) determined in 6 weeks old animals (WT in black; p22^phox^−/− in grey). P) Graph showing the ratio value of waves IV/I amplitudes as recorded following click stimuli at increasing intensities. n = 11 WT; n = 8 p22^phox^ −/−. (For interpretation of the references to color in this figure legend, the reader is referred to the Web version of this article.)

**Fig. 5:**
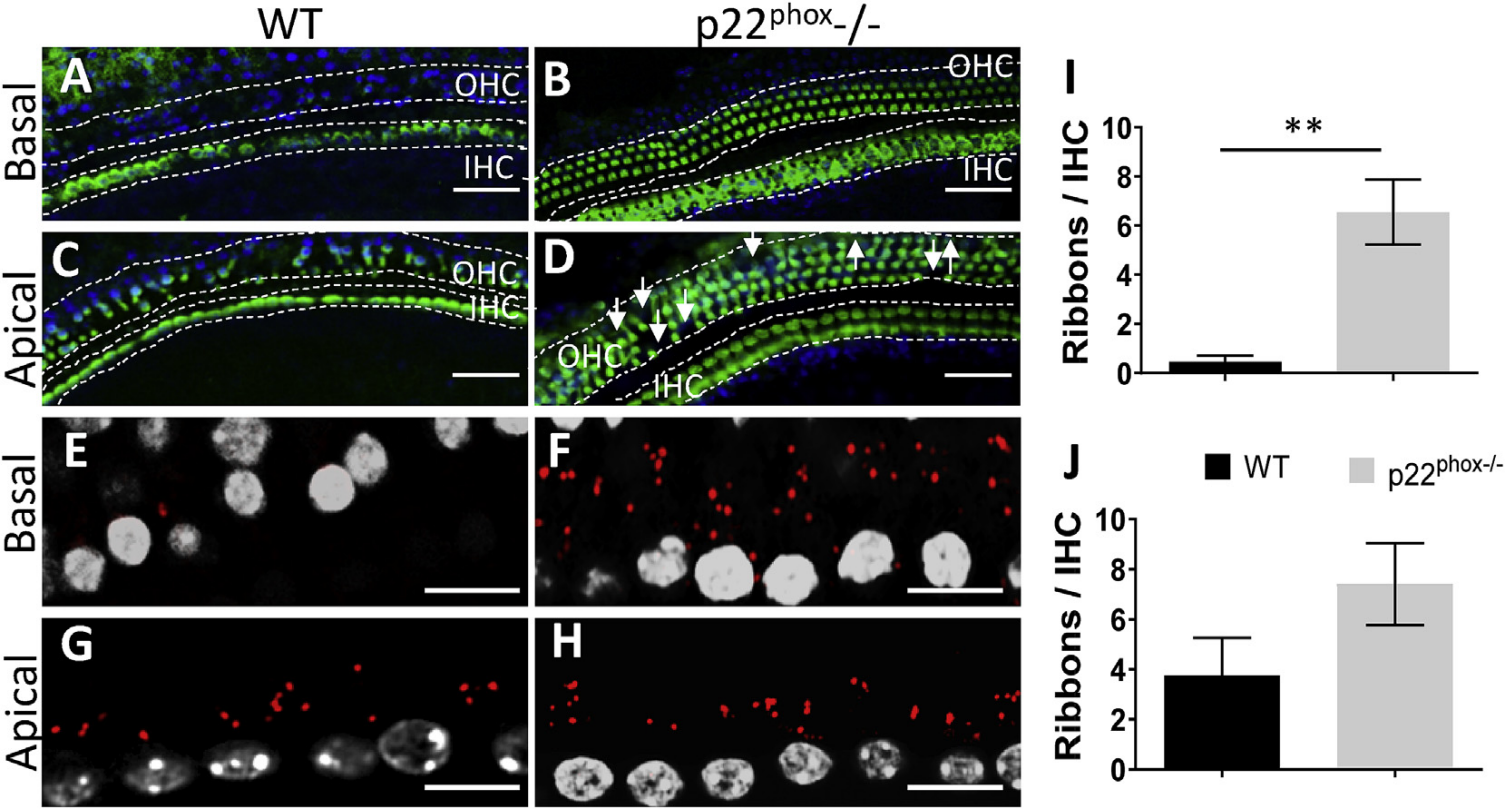
**p22**^**phox**^**deficiency prevents age-related histological alterations of the cochlea.** A-D) Representative sensory epithelium immunostaining (Myo7a in green) of (A–B) the basal and (C–D) the apical turn of the A/J WT (A and C) and p22^phox^ deficient (p22^phox−/-^) (B and D) cochleae, at the age of 26 weeks old. E-H) On the same samples, the number of synaptic ribbons between inner hair cells and spiral ganglion neurons was determined using CtBP2 immunostaining (red). E) and G) respectively, represent the basal and apical part of the cochlea of WT mice. F) and H) are respectively, the basal and apical turns of p22^phox^ deficient littermates. I-J) Bar graph showing the number of ribbons/inner hair cell (IHC). n = 3 WT; n = 3 p22phox −/−. (For interpretation of the references to color in this figure legend, the reader is referred to the Web version of this article.)

**Conserved architecture of the sensory epithelium in p22**^**phox**^ **deficient mice**. Mice were sacrificed at different ages (6, 18 and 26 weeks old) for evaluation of cochlear histomorphology (Fig. 4, Fig. 5 and Supplementary Fig. 5). In young animals (6 weeks old), approximately at the onset of hearing loss, the sensory epithelium was overall well conserved and comparable between WT and p22^phox^ animals (Fig. 4A–E). However, in contrast to p22^phox^ deficient mice, we observed a significant decrease in the presynaptic ribbons number (Fig. 4F–J) and auditory neurons density (Fig. 4K-M) in the basal turn of WT mice cochlea. On the functional level, this was accompanied by significant decrease in the ABR wave I amplitude (Fig. 4N) which was compensated by increased gain (wave IV amplitude) and modified ABR wave IV/wave I ratio (Fig. 4O–P). Aged WT mice (18 weeks old; Supplementary Figs. 5 and 26 weeks old; Fig. 5) exhibited significant loss of OHC in the basal region of the cochlea, consistent with the high frequency hearing loss (Supplementary Figs. 5A–E and Fig. 5A–D). Remarkably, architecture of the sensory epithelium of p22^phox^ deficient mice was fully preserved. In the apical turn, few missing OHC were observed in both WT and p22^phox^ deficient mice but without significant differences. Accordingly in the basal turn of the cochlea, synaptic ribbons were virtually absent in wild type mice, while they were well conserved in p22^phox^ deficient mice (Supplementary Figs. 5F–J and Fig. 5E–J). No significant differences were observed in the apex. Overall, we observed that cochlear morphological and functional changes observed in A/J mice appeared first at the synaptic/postsynaptic level, anteceding the alteration of the sensory epithelium. These damages were prevented by p22^phox^ (*Cyba*) loss of function.

**Fig. 6:**
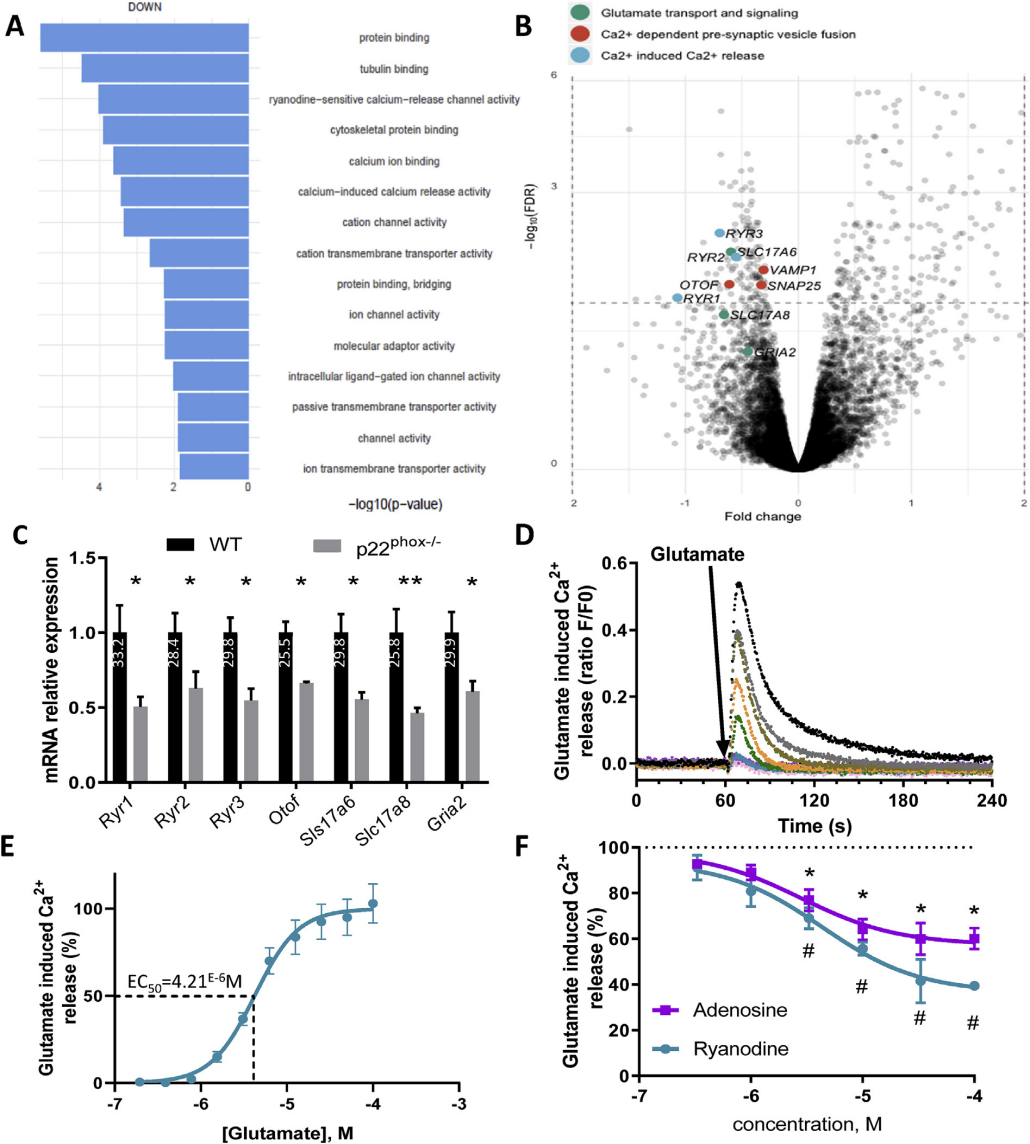
**NADPH oxidase-dependent upregulation of genes involved in the glutamatergic pathway.** RNAseq analysis was performed on 6 weeks old WT and p22^phox^ deficient cochleae. n = 3 WT; n = 3 p22^phox^−/−. A) Bar graph shows gene ontologies in the molecular function category that were downregulated in p22^phox^ deficient mice. B) RNAseq data visualized on a volcano plot reveals several downregulated genes involved in synapse and post synapse function, particularly in glutamate transport and signaling (green), Ca2+ dependent fusion of presynaptic vesicle (red) and Ca^2+^ induced Ca^2+^ release (blue). C) Bar graph showing differentially expressed genes as assessed by qPCR and expressed relatively to WT cochleae. The Ct value for the WT cochlea is indicated in white. n = 5 WT; n = 5 p22^phox^ −/− D) *Ex vivo* culture and stimulation of auditory neurons from WT A/J mice with increasing concentration of glutamate (0.1–50 μM). Before glutamate treatment, auditory neurons were loaded with Ca^2+^ sensitive ratiometric probe (FLUO-8). Traces show the kinetics of cytosolic Ca^2+^ release upon glutamate stimulation (black trace; glutamate 50 μM). E) Dose response curve of glutamate induced Ca^2+^ release allowing determination of glutamate EC50. F) Impact of pre-treatment with ryanodine or adenosine (1–100 μM) on glutamate induced Ca^2+^ release. SGN were stimulated with 5 μM glutamate, corresponding to EC90. Data represent the average±SEM of 3 independent experiments. (For interpretation of the references to color in this figure legend, the reader is referred to the Web version of this article.)

**Gene expression profiling in the cochlea from wild type and p22**^**phox**^ **deficient mice.** To further explore molecular pathways underlying the protective effect of p22^phox^ deletion in age-related hearing loss, we performed cochlear transcriptome analysis of p22^phox^ deficient mice and WT littermates (Fig. 6). We chose young animals (6 weeks) to identify early transcriptome alterations, anteceding the onset of hearing loss. Our results revealed genes associated with several molecular functions that were statistically significantly downregulated in p22^phox^ mutants (Fig. 6A). The most redundant molecular function which was lower in *CYBA* mutant mice, is related to Ca^2+^ homeostasis, and in particular ryanodine dependent Ca^2+^ release (FDR<0.05). Relevant genes include all 3 isoforms of the ryanodine receptors (*Ryr1*, *2*, *3*), involved in Ca^2+^ release from intracellular organelles, *Otoferlin*, *Vamp1* and *Snap25*, involved in Ca^2+^ dependent pre-synaptic vesicle fusion and Slc17a6, involved in glutamate transport (Fig. 6B). We also identified other genes involved in the auditory synapse function that showed a trend towards lower expression albeit not significant in the RNAseq analysis: the inner hair cells glutamate transporter (*Slc17a8*), glutamate ionotropic receptor AMPA type subunit 2 (Gria2) and Adenosine A1 receptor (*Adora1*) (data not shown). qPCR results confirmed the significantly lower expression of ryanodine receptor 1, 2, 3, otoferlin, *Slc17a6*, *Slc17a8* and *Gria2* (Fig. 6C). Except for genes associated with ribbons (presynaptic: *Vamp1, Snap25* and *Otof*), all genes were significantly enriched in the SGN (postsynaptic *Ryr1-3 Gria2, Adora1*) (Supplementary Fig. 6). Note that *Ryr3* was poorly expressed in both pre and post-synapse (Ct value of 32.6). Interestingly, downregulated genes were all centered on Ca^2+^ and glutamate signaling and might have a major role in the excitatory pathway of auditory neurons, a well-known molecular pathway of hearing loss [8].

**Analysis of the impact of adenosine and ryanodine on glutamate-induced excitation of auditory neurons**. To address the functional relevance of these genes in the excitatory response, we measured Ca^2+^ mobilization in the cytosol of auditory neurons in response to glutamate (Fig. 6D and E). Auditory neurons were obtained from spiral ganglia sphere forming stem cells as previously described [33]. While the genesis of spiral ganglia from this system has been described previously, so far no studies addressing the glutamatergic function have been performed on such a model. Upon glutamate stimulation, robust and immediate increase in cytosolic Ca^2+^ concentration was observed in the auditory neurons (Fig. 6D). This excitatory response was dose dependent and showed an EC_50_ in the micromolar range (Fig. 6E), similar to glutamate dose response curves reported in other systems [34,35]. In cortical neurons, adenosine, through adenosine receptor type I (*Adora1*), was shown to antagonize the glutamatergic pathway [36]. Interestingly, *Adora1* tended to be upregulated in mutant mice cochlea (FDR = 0.1443). To investigate the functional relevance of ryanodine receptors, we pre-incubated auditory neurons for 30 min with ryanodine, which under these conditions inhibits glutamate-induced Ca^2+^ release from the ryanodine-sensitive Ca^2+^ stores. We observed a dose-dependent inhibition of Ca^2+^ release by ryanodine with a maximal inhibition of 60%, suggesting that a substantial portion of glutamate-induced Ca^2+^ elevations in auditory neurons originates from ryanodine-sensitive Ca^2+^ stores (Fig. 6F, blue line and symbols). We next investigated the functional impact of activation of adenosine receptors. We observed a dose-dependent inhibition of cytosolic Ca^2+^ elevation by adenosine with a maximal inhibition of 40%. Thus, similar as seen in other types of neurons, adenosine receptors are inhibitors of glutamatergic cell activation stores (Fig. 6F, magenta line and symbols).

**Fig. 7:**
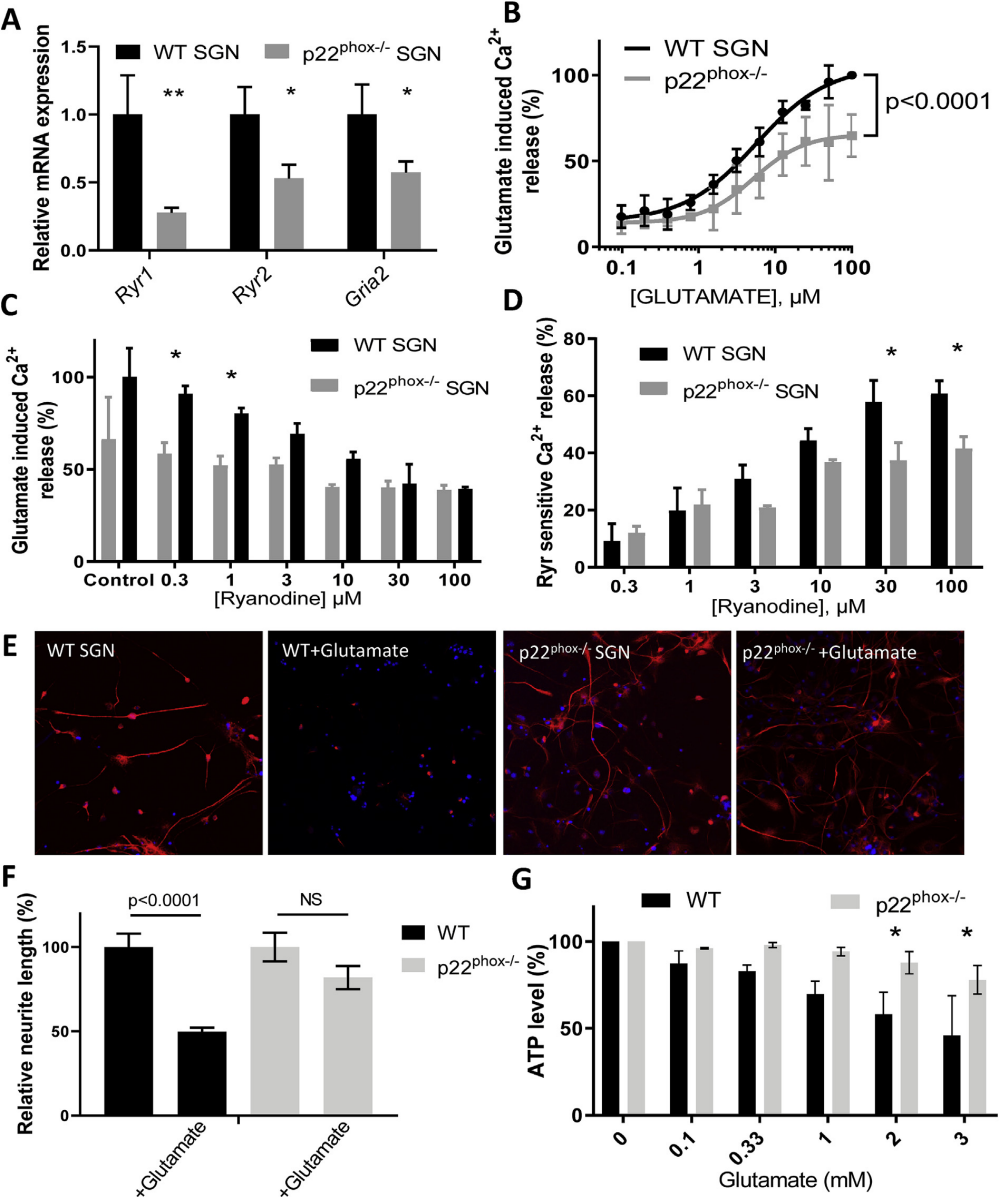
**p22**^**phox**^**deficiency mitigates glutamatergic response of auditory neurons and protects from glutamate-induced toxicity.** A) mRNA levels of Ryanodine receptor 1 (Ryr1), Ryanodine receptor 2 (Ryr2) and Glutamate ionotropic receptor AMPA type subunit 2 (Gria2) in spiral ganglion neurons from p22^phox^ deficient (grey bars) expressed relatively to WT mice (black bars, normalized to 1). n = 3 WT; n = 3 p22^phox^ −/−. B) Dose response curve of glutamate induced Ca^2+^ release in WT (black trace) and p22^phox^ −/− (grey trace) auditory neurons. p < 0.0001 with two way ANOVA. C) Effect of increasing concentrations of Ryanodine on glutamate-induced Ca^2+^ mobilization. Auditory neurons were pre-treated with ryanodine (0.3–100 μM) and stimulated with EC90 glutamate (5 μM). The bar graph shows the relative effect of ryanodine on Ca^2+^ mobilization, induced by the glutamate treatment (WT, black; p22^phox^ −/−, grey). D) The bar graph shows the proportion of glutamate stimulated Ca^2+^ mobilization inhibited by the different concentrations of ryanodine. E) High glutamate concentration impact on auditory neuron morphology. Auditory neurons from WT and p22phox-deficient A/J mice were cultured ex vivo and treated with glutamate 1 mM for 6 h. Cells were then fixed with PFA 4% and subject to BIII tubulin immunostaining (red). Nuclei were colored with DAPI (blue). Pictures are representative of 3 independent experiments. F) Bar graph showing the relative neurite length of auditory neurons treated or not with glutamate, normalized to the WT values. G) Impact of increasing glutamate concentrations on auditory neuron viability. Auditory neurons from WT or p22^phox^-deficient A/J mice were cultured ex vivo and treated with increasing concentrations of glutamate (0.1–3 mM). Viability was then assessed using an ATP measurement kit (ATP lite). Data represent the average±SEM of 3 independent experiments. (For interpretation of the references to color in this figure legend, the reader is referred to the Web version of this article.)

**Decreased Ca**^**2+**^ **induced Ca**^**2+**^ **release (CICR) in p22**^**phox**^ **deficient auditory neurons confers a protection against glutamate-mediated excitotoxicity.** To further characterize the excitatory function of p22^phox^ −/− auditory neurons, we compared the expression of key genes involved in glutamate response, namely ryanodine receptors and glutamate ionotropic receptor *Gria2* (Fig. 7A). The data demonstrate significant downregulation of ryanodine receptor 1 and 2, as well as Gria2 in p22^phox^-deficient auditory neurons (Fig. 7A). To understand the physiological relevance of this differential expression, we compared glutamate-induced Ca^2+^ release in sphere-derived auditory neurons from WT and p22^phox^−/− mice (Fig. 7B). Upon glutamate stimulation, we observed a dose-dependent increase of Ca^2+^ cytosolic concentration with a comparable EC_50_ in both WT (5.635 μM) and p22^phox^ −/− (5.183 μM) auditory neurons. However, the overall amplitude of the signal was significantly lower in p22^phox^ −/− auditory neurons (Fig. 7B; Supplementary Fig. 7). Furthermore, in absence of p22^phox^, the ryanodine-sensitive Ca^2+^ release was significantly decreased (Fig. 7C and D), consistent with the lower expression of *Ryr1* and *2* (Fig. 7A). Thus, we identified a functional role for p22^phox^ in auditory neurons, namely the control of Ca^2+^ release from ryanodine-sensitive stores. Based on the results of the gene expression analysis, as well as the functional characterization of the p22^phox^−/− and WT spiral ganglia cells, we hypothesized that the lower amplitude of the CICR in p22^phox^ deficient auditory neurons is protective against glutamate induced excitotoxicity. We therefore compared the impact of glutamate on level of cellular integrity (ATP levels) and morphology of WT and p22^phox^ −/− spiral ganglia cells (Fig. 7E–G). Upon glutamate exposure, WT auditory neurons showed dramatic morphological changes, with, in particular loss of neurites (Fig. 7E). Following glutamate exposure, neurite length in the WT cells was decreased by 50% (Fig. 7F). Remarkably, p22^phox^ deficient auditory neurons were almost fully protected from glutamate induced morphological changes with only minor decrease in average neurite length (not statistically significant). The data also showed significant decrease in ATP concentration in WT SGN in a glutamate concentration dependent manner (Fig. 7G). By contrast, the ATP content did not significantly decrease after glutamate exposure in p22^phox^-deficient cells. Together, the data demonstrate that downregulation of the excitatory pathway confers a protection against glutamate mediated toxicity in p22^phox^ deficient auditory neurons.

## 3 Discussion

Our results provide the new evidence that the absence of functional p22^phox^ in the inner ear robustly protects from age-related hearing loss and related morphological changes in mice. Mechanistically, we found that the protection is mediated by decrease of the excitatory calcium/glutamate signaling pathway in auditory neurons.

Our data provide an in-depth description of the early and progressive hearing loss in A/J mice over the first 6 months of life, beyond and in line with earlier reports [37,38]. In this background, onset of hearing loss occurs at 6 weeks of age together with predominant signs of synaptic and post-synaptic damages, including loss of auditory neurons and synaptic ribbons, as well as a decreased amplitude of ABR wave 1. Consistent with recent report in human [39], the auditory neuropathy precedes loss of hair cells, which occurs at a later stage in our model. This model mimics the progression of human presbycusis as the hearing loss in A/J mice starts in the high frequencies while lower frequencies are only affected later in life. Three mutations known to affect hearing are present in A/J mice, thereby providing structural and metabolic explanations for the severe and rapid age-related hearing loss. These include: i) a mutation in the age-related hearing loss-1 (*ahl1*) locus, which codes for cadherin 23, a structural protein of the stereocilia; ii) a mutation in *ahl4*, coding for citrate synthase, a mitochondrial enzyme of the Krebs cycle, important for ATP and NADPH synthesis and iii) mitochondrially encoded tRNA arginine, leading to a decreased efficiency in amino acid incorporation. Interestingly, a recent *in vitro* study demonstrated that decreased citrate synthase expression leads to increased production of ROS [37]. This increase of ROS was proposed to be due to a decrease in the cellular antioxidant system. However, the fact that p22^phox^ mutation can almost fully prevent the age-related hearing loss in spite of the presence of the above-mentioned mutations suggests that p22^phox^ and/or its downstream pathways are major driver of age-related hearing loss.

The A/J mouse model is a powerful tool for the study of age-related hearing loss and otoprotection. As the absence of p22^phox^ abolishes NOX1–4 catalytic activity, our data strongly support a role of NOX-derived ROS in presbycusis. However, the NOX isoform involved in hearing loss remains to be identified. Nevertheless as of today, the differential distribution of NOX isoforms in the inner ear has not been clearly elucidated. Given the problems of specificity of commercially available NOX antibodies [32], we focused our efforts on detection of NOX mRNA by qPCR and in situ hybridization providing a striking similarity of NOX isoform expression between human and mouse. The qPCR experiments confirmed high expression levels of NOX3 and p22^phox^, but also provided evidence of high levels of NOX2 and NOX4 in the cochlea. The *in-situ* hybridization experiments using the high-resolution technology RNAscope® yielded relevant new information. *Nox2* and *Nox4* mRNA appeared equally distributed throughout the cochlea. We had rather expected some strong Nox2-positive microglia cells in the region of the spiral ganglion. Note however that quiescent microglia express only low levels of Nox2, which most likely explains our observations. The most striking result was the high level of *Nox3* mRNA in the spiral ganglion cells and its virtual absence in hair cells. This finding supports a role of Nox3 in the morphological changes observed in the cochleae of 6 weeks old animals, where at the first onset of hearing loss, ribbons and SGN density are affected but not hair cells. At this point, it is not clear whether the p22^phox^-dependent degeneration of hair cells is secondary to degeneration of SGN or whether it is an independent process that occurs later in time. Concerning the first option, it is unlikely that degeneration of SGN by itself leads to loss of outer hair cells, as at least in the case of peripherin knock-out mice, SGN, but not OHCs are lost [40]. However we cannot exclude some NOX3-dependent efferent signaling mechanism that leads to a secondary OHC death. Yet, the second option, namely a delayed SGN-independent OHC death, certainly appears possible. While under physiological conditions in 6 week old mice, expression levels of NOX3 was low, it might be upregulated with aging. Indeed, there are indications that upon stress, Nox3 might be upregulated in hair cells [31]. Alternatively other p22^phox^-dependent NOX enzymes might be upregulated in the aging mice. Cell type-specific NOX knock-out will be necessary to definitively clarify this issue.

The magnitude of the protective effect of p22^phox^ deficiency is remarkable. Indeed, up to 6 month of age, there was only a minor hearing loss in p22^phox^ mutant mice, while wild type A/J mice were virtually deaf at this age. This raises the question how the lack of p22^phox^ can have such a profound effect. Indeed, NADPH oxidase are not the only source of ROS in the cochlea (e.g. mitochondria are probably important), and mechanisms other than ROS are also likely to play a role in cochlear damage [8]. Thus, p22^phox^ may represent “the straw that breaks the camel's back”, i.e. the cumulative effect of many insults leads to hearing loss and by removing one of them (i.e. p22^phox^), the function of the cochlea is maintained.

Our results demonstrate that p22^phox^ loss of function is strikingly important in sensory neurons as it leads to a lower expression of genes of the neuronal excitatory pathway in the cochlea. This supports a key role of NOX-derived ROS in this mechanism. Downregulated genes harbor functions in Ca^2+^ signaling (ryanodine receptors 1,2,3), in synaptic transmission (Otoferlin, *Snap25* and *Vamp1*), as well as in the glutamatergic pathway (*Slc17a6*, *Slc17a8* and *Gria2*). The cytosolic free Ca^2+^ concentration plays a key role in pre- and post-synaptic auditory signal transmission. On the presynaptic side, Ca^2+^ influx through Ca^2+^ channels activates synaptic vesicle fusion and subsequent glutamate release [[41], [42], [43]]. On the post-synaptic side, glutamatergic transmission involve the glutamate ionotropic AMPA type subunit 2 (*Gria2*) leading to Ca^2+^ increase in auditory neurons [44]. The lower expression of neuronal excitatory genes may be explained by a delay in development as previously described [45] or to a downregulation of these genes in p22^phox^ mutant mice. Interestingly, lower expression levels of ryanodine receptors in p22^phox^ mutant mice are sufficient for normal hearing. However, our data also suggest that enhanced expression of ryanodine receptors leads to glutamate-induced toxicity and subsequent hearing loss, which was mitigated in p22^phox^ loss of function mice. In our *in vitro* auditory neuron model, we provide direct evidence that ryanodine receptors are involved in the post-synaptic Ca^2+^ signal: glutamate-induced Ca^2+^ elevations are inhibited by ryanodine in a dose-dependent manner. From the relative inhibition of the Ca^2+^ signal, we can conclude that ryanodine receptors are important mediators of the excitatory signal in the cochlea at the post synaptic level. This observation is consistent with previous findings that AMPA-type glutamate receptors and intracellular Ca^2+^ stores are coupled via RyR-dependent CICR in primary auditory neurons [46]. Furthermore, intracochlear perfusion of ryanodine in guinea pigs in vivo has been shown to decrease auditory nerve compound action potentials, indicating the importance of RyRs in cochlear transduction [47]. Direct activation of Ryr2 by ROS is well-documented [48,49], however, in the present study, transcriptomic data suggest that NOX-derived ROS rather act at the transcriptional level. Further characterization of the promoters and the transcriptional machinery will be required to understand whether specific redox-sensitive transcription factors are involved.

To explore key finding of our study, namely a protection of p22^phox^-deficient animals from age-related hearing loss, other findings need to be discussed. The A1AR adenosine receptor has been shown to be linked to hearing loss and NOX signaling: mice deficient for the A1AR exhibit greater susceptibility to noise exposure [50] and A1AR activation confers protection against cisplatin ototoxicity through downregulation of NOX3 [51]. Our results show only a trend towards increased levels of A1AR in p22^phox^-deficient mice (p = 0.0064; FDR = 0.1443). Interestingly, A1AR is an inhibitor of the excitatory pathway [52,53], and in our hands, adenosine significantly decreased the excitatory signal in auditory neurons (Fig. 6). This might provide an additional element for the understanding why p22^phox^-deficient mice are protected.

To the best of our knowledge, this is the first study investigating age-related hearing loss in p22^phox^ deficient mice. However, the impact of Nox3 deletion has respectively been addressed in the context of cisplatin [31] and noise [38] induced hearing loss. The first study reports a protective impact in cisplatin-induced hearing loss following injection of Nox3 siRNA in the middle ear while the second publication by Lavinsky and collaborators using Nox3 mutant mouse concluded that Nox3 might be protective against noise-induced inner ear damage. In terms of pattern of hearing loss, the Lavinsky study did not show an impact of Nox3 deficiency on noise-induced hearing loss at high frequencies, but a small protective effect of Nox3 at low frequencies, statistically significant only at 8 kHz. Our results studying age-related hearing loss showed a protective effect of p22^phox^ deficiency at frequencies above 8 kHz. The reason for these apparently conflicting data may be due to i) differences in the type of hearing loss (noise-induced, ototoxicity and age-related), ii) mice in a different genetic background were studied (black6 vs A/J), and iii) importantly, a different type of NOX-deficient mouse was investigated (NOX3-deficiency vs. p22^phox^ deficiency, corresponding to a functional NOX1-4 knock-out). Future studies addressing each isoform regulated by p22^phox^ may provide a better understanding of such differences.

Our study provides first conclusive evidence that p22^phox^ is key regulator of age-related hearing loss, most likely through regulation of the activity of ROS-generating NADPH oxidases. The extent of the protection of p22^phox^-deficient animals is astonishingly strong. Our data suggest that p22^phox^ – master regulator of the NOX pathway – is not only regulating the levels of non-specific ROS-induced damage in the cochlea. In fact, we demonstrate that level of expression of genes of the excitatory pathway is strikingly diminished in p22^phox^ mutant mice. Thus, our data suggest that NOX-enhanced excitotoxicity contributes to age-related hearing loss. We believe that targeting NOX catalytic activity is a promising strategy to prevent age-related hearing loss. Small molecules NOX inhibitors would be interesting tools, but might be difficult to target to the inner ear. Local delivery of molecular therapies (i.e. RNA knock-down of p22^phox^) may provide long term protection against presbycusis and other related inner ear damage, but will equally have to be precisely targeted [54].

## 4 Methods

### 4.1 Animal procedures

A.B6 *Tyr*^+^-*Cyba*^*nmf333*^/J mice were purchased from Jackson lab (strain 005445) and colonies were maintained at the animal facility of the University of Geneva through heterozygous x heterozygous mating, ensuring equal proportion of WT and p22^phox^ deficient littermates. Both male and female, were subjected to hearing threshold determination using the auditory brainstem response at different time points from 4 weeks old to 26 weeks old. At the end of the experiments, animals were sacrificed for cochlea histology or mRNA extraction. For the auditory tests, or before sacrifice, mice were anesthetized with a mix of Ketamine (100 mg/kg) and Xylazine (10 mg/kg).

### 4.2 Auditory brainstem response

Animals were anesthetized by intraperitoneal injection Ketamine 100 mg/kg and of Xylazine 5 mg/kg, and placed upon a heating pad to maintain body temperature. Depth of anesthesia was tested every 30 min by testing the pedal withdrawal reflex. If necessary, additional injections of about 50% of the initial dose were given. ABR recordings were performed in a sound proof chamber (IAC Acoustics, Illinois IL, USA). For stimulus generation and recording of responses, a multi-function IO-Card (National Instruments, Austin TX, USA) was used, housed in an IBM compatible computer. An integrated software package for stimulus generation and recording (Audiology_lab; Otoconsult, Frankfurt, Germany) was used. Sound pressure level was controlled with an attenuator and amplifier (Otoconsult, Frankfurt, Germany). Stimuli were delivered to the ear in a calibrated open system by a loudspeaker (AS04004PR-R, PUI Audio, Inc., Dayton, USA) placed 3 cm lateral to the animals’ pinna. Sound pressure was calibrated on-line prior to each measurement with a microphone probe system (Bruel&Kjaer 4191) placed near the animals’ ear. Recorded signals were amplified and bandpass filtered (80 dB; 0.2–3.0 kHz) using a filter/amplifier unit (Otoconsult, Frankfurt, Germany). For the recordings, silver electrodes were placed subcutaneously on the mouse forehead (+), on the mastoid of the recorded ear (−) and a reference electrode on the back. ABR were recorded, following stimulation with 100μs clicks or 3 ms tone pipes (2.0–45.2  kHz at a resolution of 2 steps per octave). For all frequencies, ABRs were recorded from 0 to 90 dB SPL in 3 dB steps. Electrical signals were averaged over 256 repetitions of stimulus pairs with alternating phase. Hearing thresholds were defined as the sound pressure level where a stimulus-correlated response was clearly identified by visual inspection of the averaged signal.

### 4.3 Cochlea histology (mid modiolar cut, cytocochleograms and ribbons staining)

At the end of the last ABR measurement, anaesthetized mice were sacrificed by cervical dislocation followed by decapitation. Temporal bones were isolated from the skull in order to extract the inner ears, and after proper dissection cochlea were placed in 4% paraformaldehyde overnight at room temperature. Cochleae were decalcified using USEDECALC solution (Medite commercial solution) under sonication for 48 h (following ultrasonic technique of USE 33 decalcification machine from Medite, Cat. No. 03-3300-00). Finally, decalcified cochlea were microdissected to perform cytocochleograms and immunohistochemistry, or mid modiolar cuts (embedded in paraffin) with Hematoxilin-Eosin staining. As general rule, one ear from each animal was used for cytocochleograms and the other ear for mid-modiolar staining.

#### 4.3.1 Immunohistochemistry and confocal microscopy (cytocochleograms)

Decalcified cochleae were microdissected with thin forceps under binocular microscope. The bony shell was removed to expose the Organ of Corti (OC), followed by the removal of the stria vascularis and separation of the sensory epithelium from the spiral ganglion. The basal and middle turns of the OC were cut in two pieces each one and the apical turn was preserved as one piece, resulting in 5 pieces transferred into 400 μL PBS solution in a 48 well plate. Samples were then permeabilized (3% Triton-X 100 in PBS 1X) for 30 min at room temperature and immersed in a blocking buffer, containing 2% bovine serum albumin (BSA) and 0.01% Triton-X 100 in PBS, for 1 h at room temperature. Explants were incubated with anti-MyoVIIa (1:200, rabbit; Proteus, USA) and anti-CtBP2 (1:200, monoclonal mouse, BD Bioscience) antibodies in blocking buffer overnight at 4 °C. On the following day, tissues were rinsed three times with PBS and incubated, for 2 h at room temperature, with the secondary antibodies anti-rabbit Alexa Fluor 488 (1:500; Invitrogen, USA) and goat anti-mouse Alexa 555 (1:500; Life Technologies) diluted in blocking buffer. Explants were washed 3 times with PBS and mounted on a glass slide with Fluoroshield containing DAPI (Sigmaaldrich, USA). The labelled cells were visualized with a confocal laser-scanning microscope (Zeiss LSM700) equipped with a CCD camera (Leica Microsystems) employing the Plan-Neofluar 20X/0.50 and Plan-Apochromat 63X/1.4 (Oil) objectives. Hair cell and ribbons counting was performed using image J software.

#### 4.3.2 Mid modiolar hematoxylin eosin staining

Decalcified cochlea were dehydrated and embedded in paraffin. Mid-modiolar cuts of 5 μm were processed and loaded onto gelatin-coated slides. Harris’ Hemalun/eosin staining was performed on the slides. The samples were qualitatively analyzed for density of spiral ganglia using image J software.

### 4.4 Quantitative analysis of cochlea morphology

The SGN density was determined by nuclear counting, employing the hematoxylin-eosin stained slides, of the Rosenthal's canal at each different cochlear turn (apical, medial and basal). Five non-consecutive sections were evaluated. To normalize the data, the total neuronal amount was divided between each Rosenthal's area (in mm^2^), and the measured densities were averaged. For the synaptic ribbons count, 10-15 μm Z stack (0.7 μm steps) were performed with the 63X objective and merged in a single picture, to ensure a proper counting of the ribbons distributed along the Z axis. For each section of the OC, ribbons belonging to two different segments of 10–15 inner hair cells were counted to obtain an average of each cochlear turn. Additionally, 20X pictures were employed to evaluate hair cells (HC) survival (cytocochleogram). Inner hair cells (IHC) and outer hair cells (OHC) population were recorded in two representative areas of 100 μm from each cochlear section, in order to average the HC survival rate of each turn (apical, medial and basal). The open source image J program was used for the image analysis.

### 4.5 Gene expression analysis

#### 4.5.1 Mouse cochlea RNA extraction

Cochlea were quickly dissected in cold PBS, carefully removing remaining blood and surrounding tissues, immediately frozen in liquid nitrogen and kept at −80 °C for further RNA extraction. Cochlea were physically homogenized with clean steel balls and tissueLyser (Qiagen) for 30 s at 30 rpm, and the DNA and RNA precipitated with 750 μl Trizol. After adding 150 μl chloroform, (shaking, resting and centrifuging step) we collected the aqueous phase and performed column purification with Qiagen RNeasy Micro kit, ignoring the lysis and homogenization steps described in the kit protocol (adding directly 70% ethanol) (adapted from Ref. [55]).

#### 4.5.2 Human foetal cochlea RNA extraction

The inner ear was isolated from aborted human fetuses ranging from W8 to W12 post conception. Procurement and procedures were performed with full approval by the Ethics Committee of the State Geneva, Switzerland and following signed informed consent of the donors. The postmenstrual date was used for the calculation of the fetal stage. Tissue dissection was performed in ice-cold Hanks’ balanced salt solution (HBSS). The rest of the procedure was similar as for mouse cochlea RNA extraction (see paragraph above).

#### 4.5.3 Real time quantitative polymerase chain reaction

Following the cochlear extraction, RNA concentration was determined using a Nanodrop. 500 ng of RNA was used for cDNA synthesis using the Takara PrimeScript RT reagent Kit, following manufacturer's instruction. Real-time PCR was performed using SYBR green assay on a 7900HT SDS system from ABI. The efficiency of each primer was verified with serial dilutions of cDNA. Relative expression levels were calculated by normalization to the geometric mean of the three house-keeping genes (*Eef1a*, *Tubb* and *Actb* for mouse and *EEF1a*, *B2M* and *GAPDH* for human samples). The highest normalized relative quantity was arbitrarily designated as a value of 1.0. Fold changes were calculated from the quotient of means of these RNA normalized quantities and reported as ±SEM. Sequences of the primers used are provided in Supplementary Table 1.

#### 4.5.4 RNAscope® (ACDbio, bio-techne, minneapolis, Minnesota, United States)

Cochleae were fixed, decalcified and embedded in paraffin (see above - mid modiolar cuts). 5 μm mid modiolar slices were loaded onto gelatin-coated slides and subjected to RNAscope probe hybridization. RNAscope Multiplex Fluorescent V2 assay (Bio-techne, Cat. No. 323110) or RNAscope 2.5 HD Assay – BROWN assay (Bio-techne, Cat. No. 322310) was performed according to manufacturer's protocol, paraffin sections were hybridized with the probes Mm-Nox3-C1 (Bio-techne, Cat. No. 481989), Mm-Ppib-C1 (Bio-techne, Cat. No. 313911) as positive control and DapB-C1 (Bio-techne, Cat. No. 310043) as negative control at 40 °C for 2 h and revealed with TSA Opal570 (PerkinElmer, Cat. No. FP1488001KT). For Fluorescent assay, tissues were counterstained with DAPI and mounted with FluoromountG (Bioconcept, Cat. No. 100.01). For BROWN assay, Tissues were counterstained with Mayer hematoxyline for 30 s, dehydrated, cleared and mounted with Tissue-Tek® Glas™ (Sakura, Cat. No. 1408). Samples were visualized with a confocal laser-scanning microscope (Zeiss LSM700) equipped with a CCD camera (Leica Microsystems) employing the Plan-Neofluar 20X/0.50 and Plan-Apochromat 63X/1.4 (Oil) objectives. Pictures were analyzed using image J software.

#### 4.5.5 RNA seq

TruSeq ribodepleted stranded mRNA from 6 weeks old A/J mice (3 WT vs. 3 p22^phox^ deficient) was applied to eliminate ribosomic RNA and sequenced using Illumina TruSeq protocol. The sequencing quality control was done with FastQC v.0.11.5. The sequences were mapped with the TopHat v.2.0.11 software to the UCSC mm10 mouse reference. The biological quality control and summarization were performed using the PicardTools v.1.141. The table of counts with the number of reads mapping each gene feature of UCSC mm10 was prepared with HTSeq v0.6p1. The differential expression analysis was performed with the statistical analysis R/Bioconductor package edgeR v. 3.14.0. Briefly, the counts were normalized according to the library size and filtered. The genes having a count above 1 count per million reads (cpm) in at least 4 samples were kept for the analysis. The initial number of genes in the set was 23′420 and after the poorly or non-expressed genes were filtered out, 15′126 genes were left. The p values of differentially expressed gene analysis were corrected for multiple testing problems using the Benjamini-Hochberg (BH) procedure. All RNA-sequencing data files were submitted to the arrayexpress database at EMBL-EBI (www.ebi.ac.uk/arrayexpress) under the accession number E-MTAB-8668.

### 4.6 In vitro culture of mouse SGN

Mouse spiral ganglion neurons were isolated from p2-p5 A/J pups, cultured and passaged as previously described [33]. Briefly, neurospheres from spiral ganglia were maintained in DMEM:F12 with 1x N2 and B27 supplement in presence of bFGF, IGF1, Heparan sulfate and EGF. For passages or differentiation, cells were dissociated with acutase and mechanically disintegrated. Following filtration with a 70 μm filter, retaining non dissociated spheres, SGN were counted and plated in a 384 or 96 well plate coated with matrigel (hESC qualified, Corning, New York, USA) with medium without growth factors, in order to induce differentiation. After 5 days of differentiation, cells were employed for cytosolic Ca2+ measurement and excitotoxicity assay.

### 4.7 Measurement of Ca^2+^ cytosolic release

16000 cells/well were differentiated to SGN on a 96 well plate coated with matrigel, as described above (*in vitro* culture of spiral ganglion neurons). Differentiated cells were loaded with FLUO-8 (Interchim, Montluçon, France) according to the manufacturer protocol. After 45min incubation at 37 °C, the glutamate-induced cytosolic calcium release was assessed in a FDSS/μCELL Functional Drug Screening System (Hamamatsu, Yokohama, Japan). The neuronal kinetics of calcium release was followed over 10min following glutamate addition, with 1 measure every 0.5 s. The impact of ryanodine or adenosine (0-100 μM), respectively added 30min or 24 h prior glutamate, was also investigated.

### 4.8 Impact of high glutamate concentration on auditory neuron ATP content

16000 cells/well were differentiated to SGN on a 96 well plate coated with matrigel, as described above (*in vitro* culture of spiral ganglions). Differentiated cells were then treated with increasing concentrations of glutamate for 6 h and the SGN viability was assessed using the ATPLite kit (PerkinElmer, Wellesley, USA), following manufacturer's instructions using spectra L reader.

### 4.9 Impact of high glutamate concentration on auditory neuron morphology

Auditory neuron progenitors were seeded on matrigel coated coverslips and differentiated into auditory neurons for 5 days. Cells were then exposed to 1 mM glutamate for 6 h and fixed for 10 min with PFA 4%. Following the fixation, neurons were permeabilized (0.2% Triton-X100 in PBS 1X) for 30 min at room temperature and immersed in a blocking buffer containing 2% BSA and 0.01% Triton-X 100 for 1 h at room temperature. SGNs were incubated with the primary antibody anti-BIII tubulin (1:1000, mouse; Biolegend, USA) in blocking buffer overnight at 4 °C. On the following day, tissues were rinsed three times with PBS and incubated with the secondary antibody anti-mouse Alexa Fluor 555 (1:500; Invitrogen, USA) in blocking buffer for 2 h at room temperature. Coverslips were washed 3 times with PBS and mounted on a glass slide with Fluoroshield containing DAPI (Sigmaaldrich, USA). The labelled cells were visualized with a confocal laser-scanning microscope (Zeiss LSM700) equipped with a CCD camera (Leica Microsystems) with Plan-Neofluar 10X/0.30 NA objective. Neurite length evaluation was performed using image J software.

### 4.10 Statistics

All data were analyzed using Two-way ANOVA followed by Bonferroni multiple comparison test using GraphPad Prism software unless where stated otherwise in the Figure legend. Values with p < 0.05 was considered as statistically significant. *; p < 0.05, **p < 0.01, ***; p < 0.005, ****p < 0.0005.

### 4.11 Study approval

All procedures were approved by the local veterinary office and the Commission for Animal experimentation of the Canton of Geneva, Switzerland, authorization number GE/106/18.

## Author contributions

FR, KHK and PS designed the study; FR, MC, GNS, AM, VBCK, MP and YC acquired the data; FR, GNS, YC, VJ, KHK and PS analyzed the data; FR, VJ, KHK and PS wrote the manuscript.

## Declaration of competing interest

The authors have declared that no conflict of interest exists.
