# Supplementary material for: Redox activation of excitatory pathways in auditory neurons as mechanism of age-related hearing loss
Source: Redox Biol. 2020 Jan 20;30:101434. doi: 10.1016/j.redox.2020.101434 (PMC7016250; doi:10.1016/j.redox.2020.101434)
Supplement: Multimedia component 1 [file mmc1.pptx]

## Slide 1
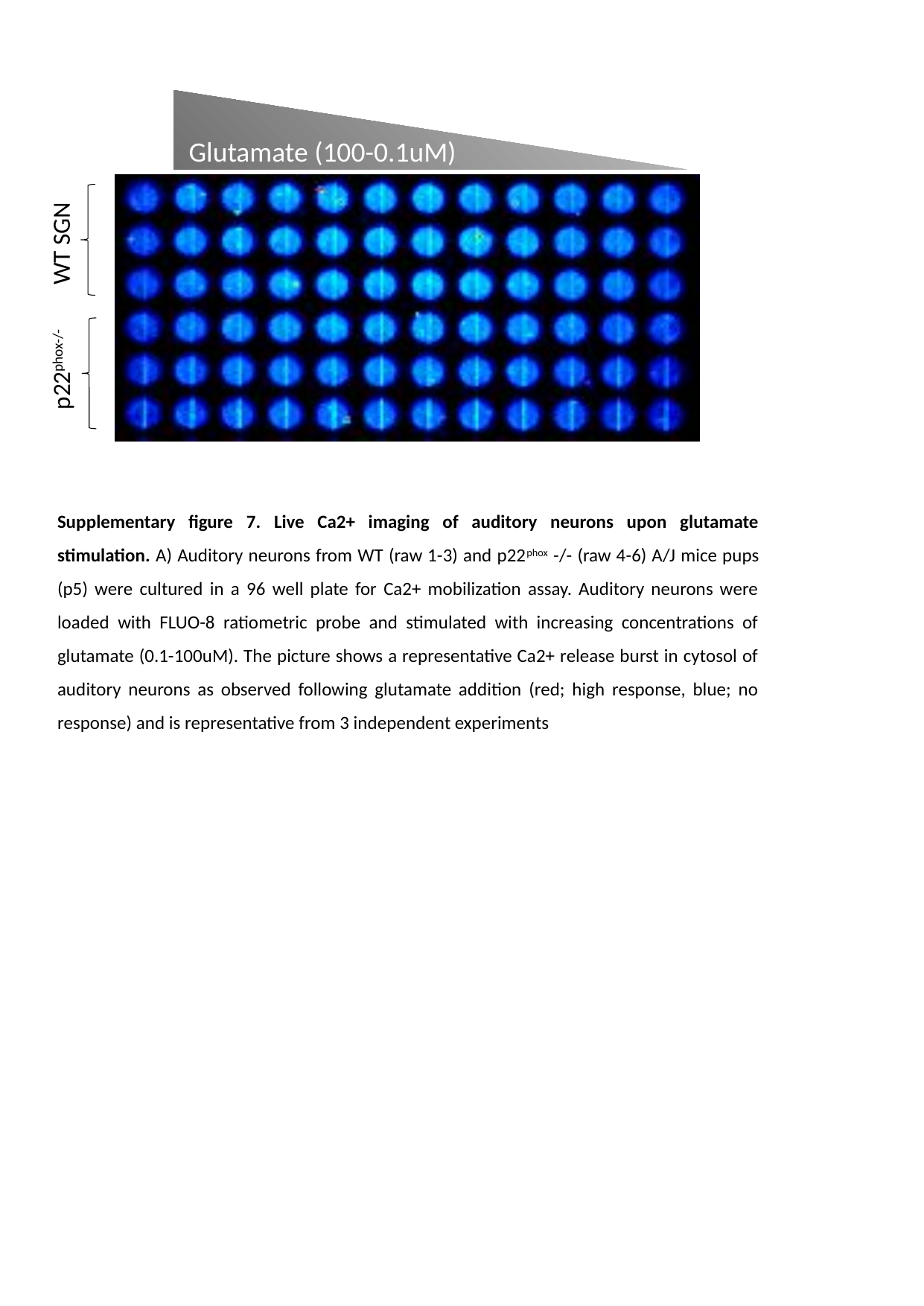

Glutamate (100-0.1uM)
WT SGN
p22phox-/-
Supplementary figure 7. Live Ca2+ imaging of auditory neurons upon glutamate stimulation. A) Auditory neurons from WT (raw 1-3) and p22phox -/- (raw 4-6) A/J mice pups (p5) were cultured in a 96 well plate for Ca2+ mobilization assay. Auditory neurons were loaded with FLUO-8 ratiometric probe and stimulated with increasing concentrations of glutamate (0.1-100uM). The picture shows a representative Ca2+ release burst in cytosol of auditory neurons as observed following glutamate addition (red; high response, blue; no response) and is representative from 3 independent experiments
