# Supplementary material for: Redox activation of excitatory pathways in auditory neurons as mechanism of age-related hearing loss
Source: Redox Biol. 2020 Jan 20;30:101434. doi: 10.1016/j.redox.2020.101434 (PMC7016250; doi:10.1016/j.redox.2020.101434)
Supplement: Multimedia component 2 [file mmc2.pptx]

## Slide 1
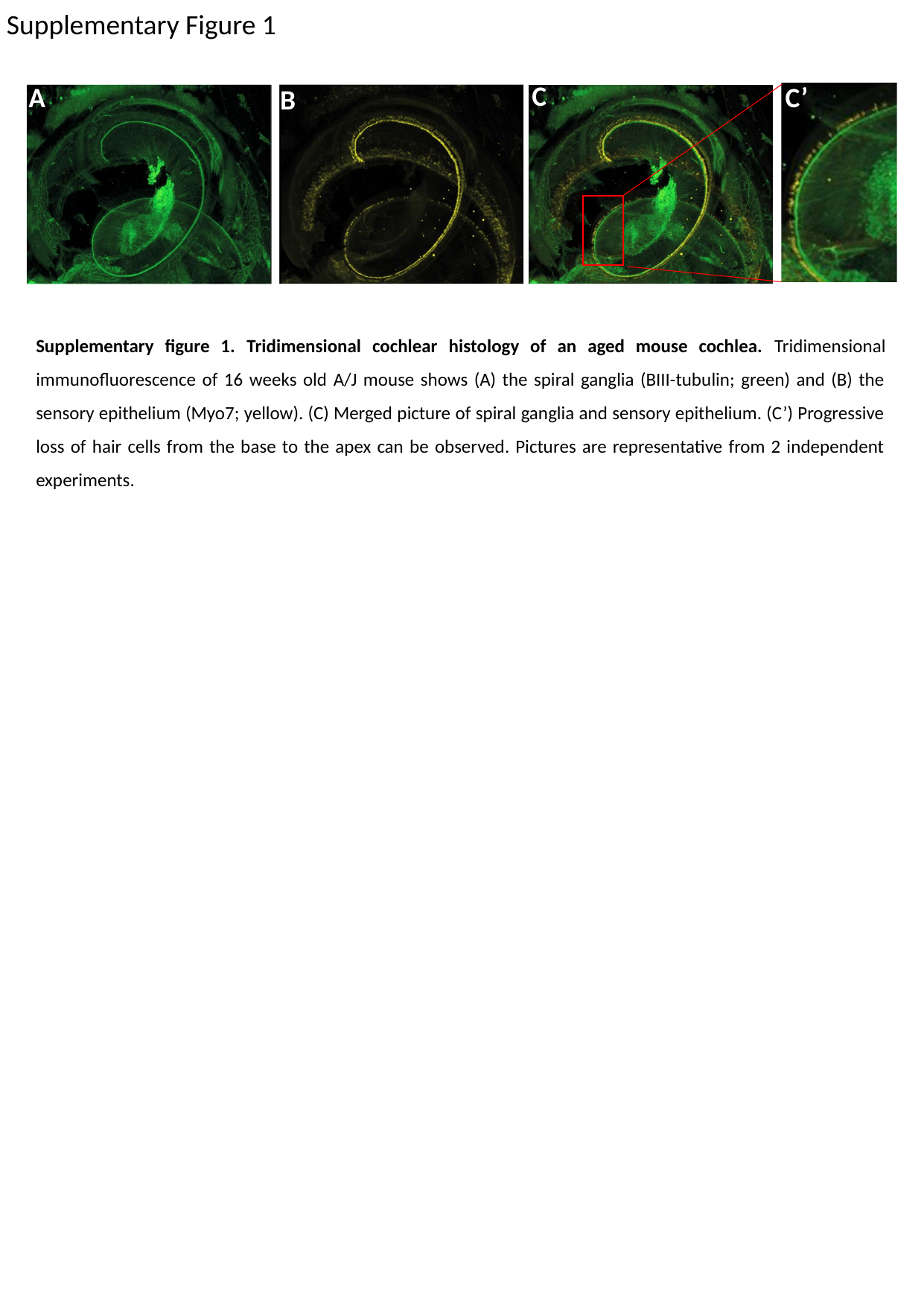

Supplementary Figure 1
C
C’
A
B
Supplementary figure 1. Tridimensional cochlear histology of an aged mouse cochlea. Tridimensional immunofluorescence of 16 weeks old A/J mouse shows (A) the spiral ganglia (BIII-tubulin; green) and (B) the sensory epithelium (Myo7; yellow). (C) Merged picture of spiral ganglia and sensory epithelium. (C’) Progressive loss of hair cells from the base to the apex can be observed. Pictures are representative from 2 independent experiments.

## Slide 2
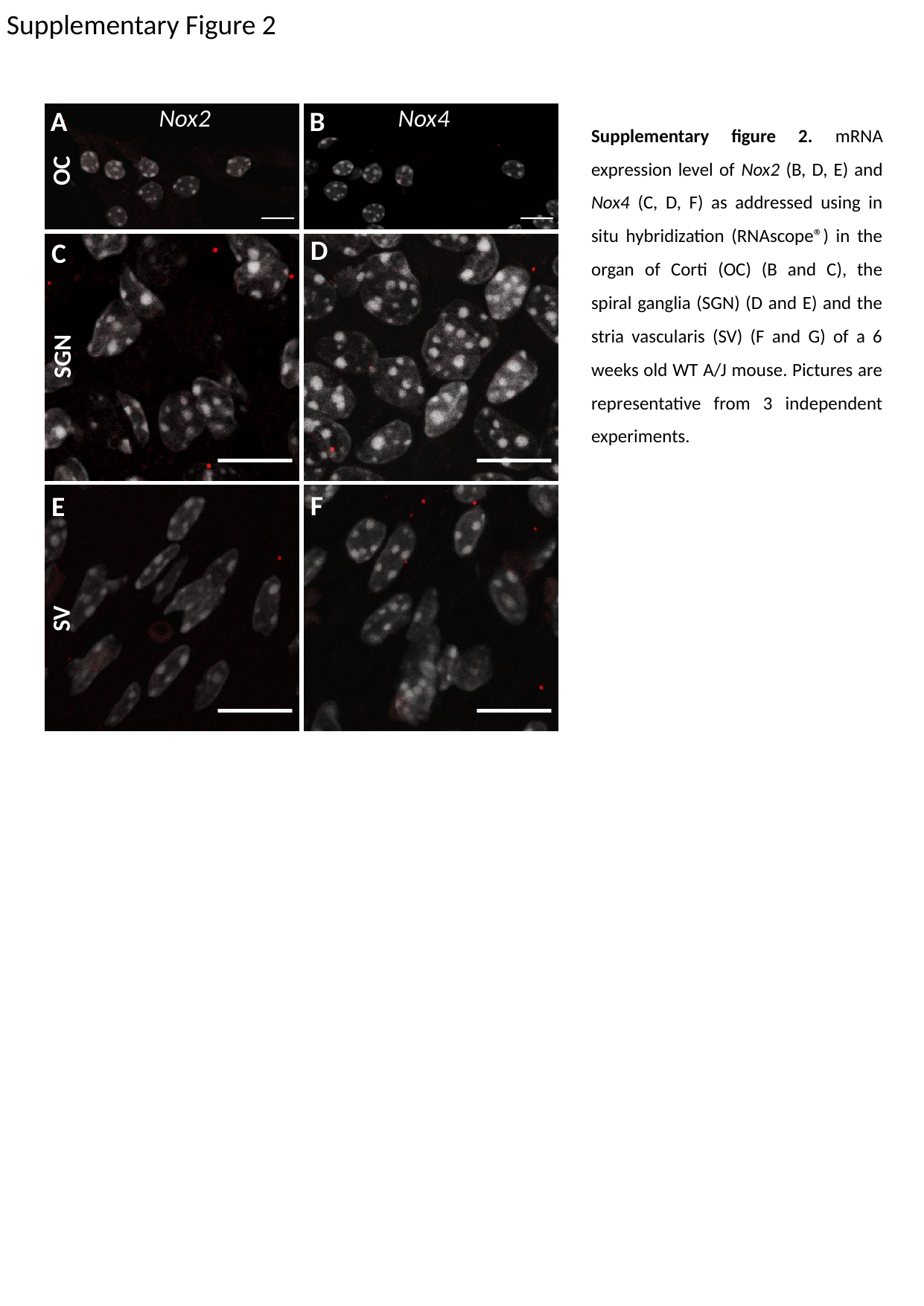

Supplementary Figure 2
Nox2
Nox4
B
A
D
C
F
E
OC
SGN
SV
Supplementary figure 2. mRNA expression level of Nox2 (B, D, E) and Nox4 (C, D, F) as addressed using in situ hybridization (RNAscope®) in the organ of Corti (OC) (B and C), the spiral ganglia (SGN) (D and E) and the stria vascularis (SV) (F and G) of a 6 weeks old WT A/J mouse. Pictures are representative from 3 independent experiments.

## Slide 3
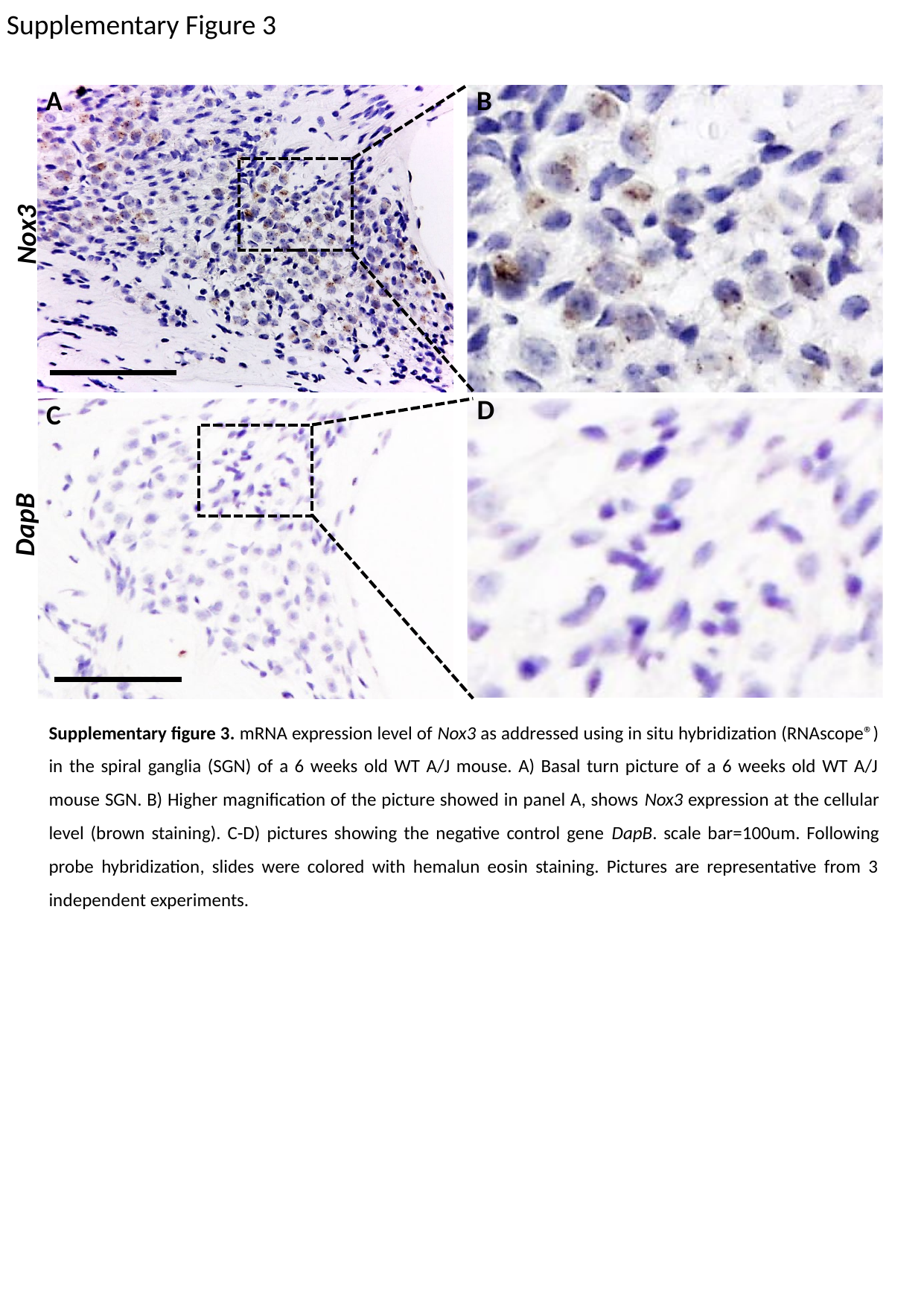

Supplementary Figure 3
A
B
Nox3
D
C
DapB
Supplementary figure 3. mRNA expression level of Nox3 as addressed using in situ hybridization (RNAscope®) in the spiral ganglia (SGN) of a 6 weeks old WT A/J mouse. A) Basal turn picture of a 6 weeks old WT A/J mouse SGN. B) Higher magnification of the picture showed in panel A, shows Nox3 expression at the cellular level (brown staining). C-D) pictures showing the negative control gene DapB. scale bar=100um. Following probe hybridization, slides were colored with hemalun eosin staining. Pictures are representative from 3 independent experiments.

## Slide 4
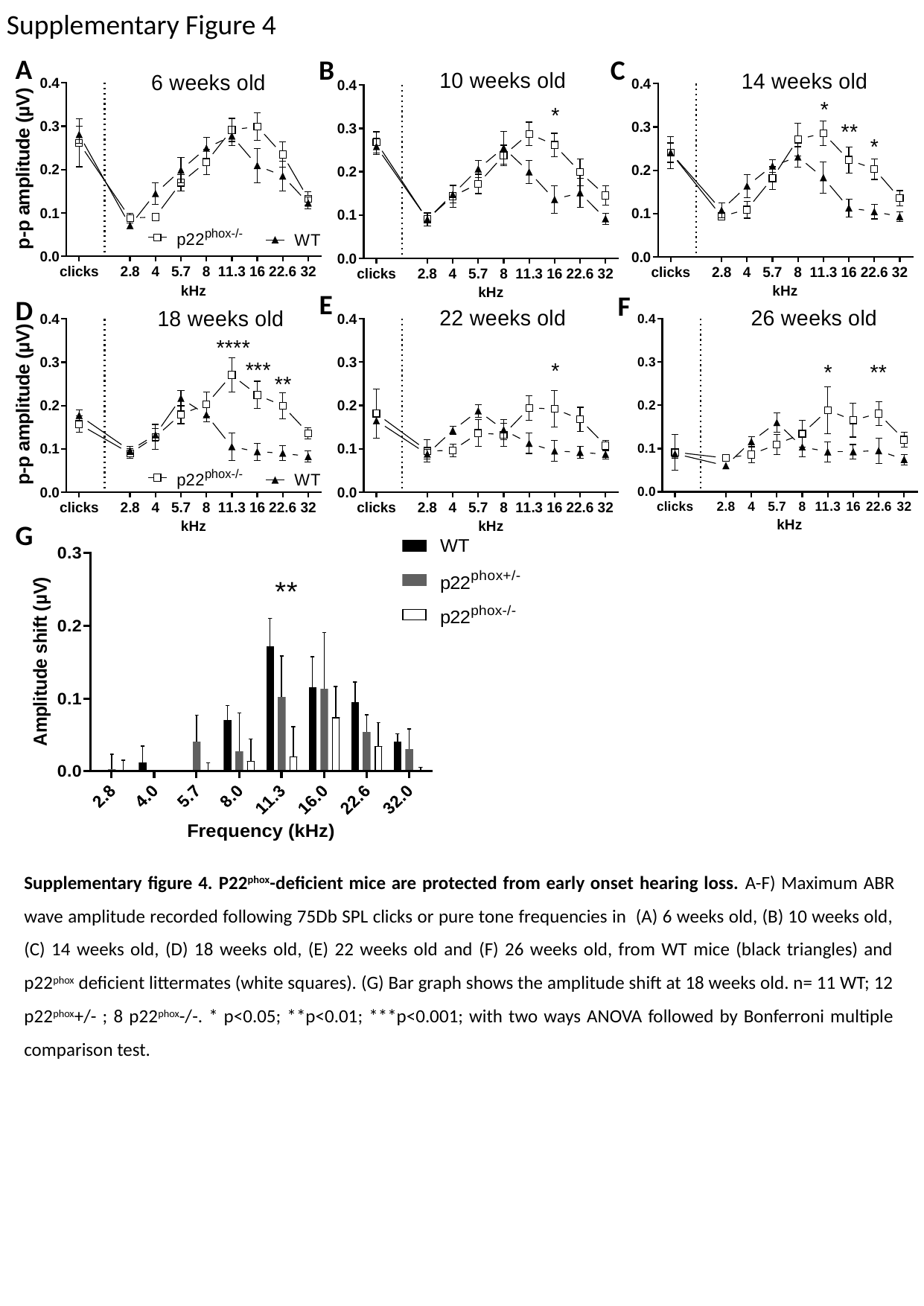

Supplementary Figure 4
a
b
c
e
f
d
g
Supplementary figure 4. P22phox-deficient mice are protected from early onset hearing loss. A-F) Maximum ABR wave amplitude recorded following 75Db SPL clicks or pure tone frequencies in (A) 6 weeks old, (B) 10 weeks old, (C) 14 weeks old, (D) 18 weeks old, (E) 22 weeks old and (F) 26 weeks old, from WT mice (black triangles) and p22phox deficient littermates (white squares). (G) Bar graph shows the amplitude shift at 18 weeks old. n= 11 WT; 12 p22phox+/- ; 8 p22phox-/-. * p<0.05; **p<0.01; ***p<0.001; with two ways ANOVA followed by Bonferroni multiple comparison test.

## Slide 5
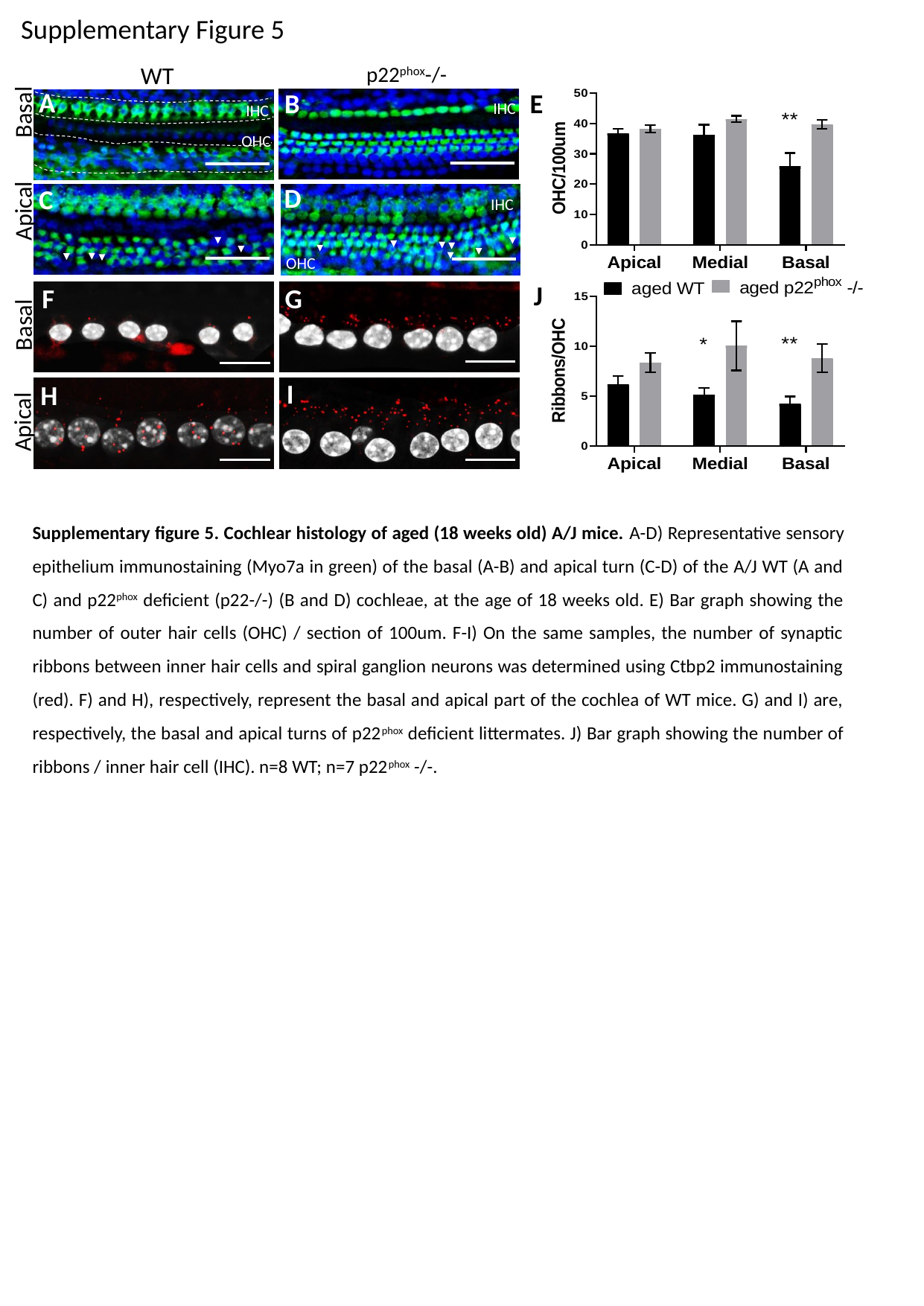

Supplementary Figure 5
p22phox-/-
WT
Basal
a
b
E
IHC
IHC
OHC
D
C
IHC
Apical
OHC
F
G
Basal
I
H
J
Apical
Supplementary figure 5. Cochlear histology of aged (18 weeks old) A/J mice. A-D) Representative sensory epithelium immunostaining (Myo7a in green) of the basal (A-B) and apical turn (C-D) of the A/J WT (A and C) and p22phox deficient (p22-/-) (B and D) cochleae, at the age of 18 weeks old. E) Bar graph showing the number of outer hair cells (OHC) / section of 100um. F-I) On the same samples, the number of synaptic ribbons between inner hair cells and spiral ganglion neurons was determined using Ctbp2 immunostaining (red). F) and H), respectively, represent the basal and apical part of the cochlea of WT mice. G) and I) are, respectively, the basal and apical turns of p22phox deficient littermates. J) Bar graph showing the number of ribbons / inner hair cell (IHC). n=8 WT; n=7 p22phox -/-.

## Slide 6
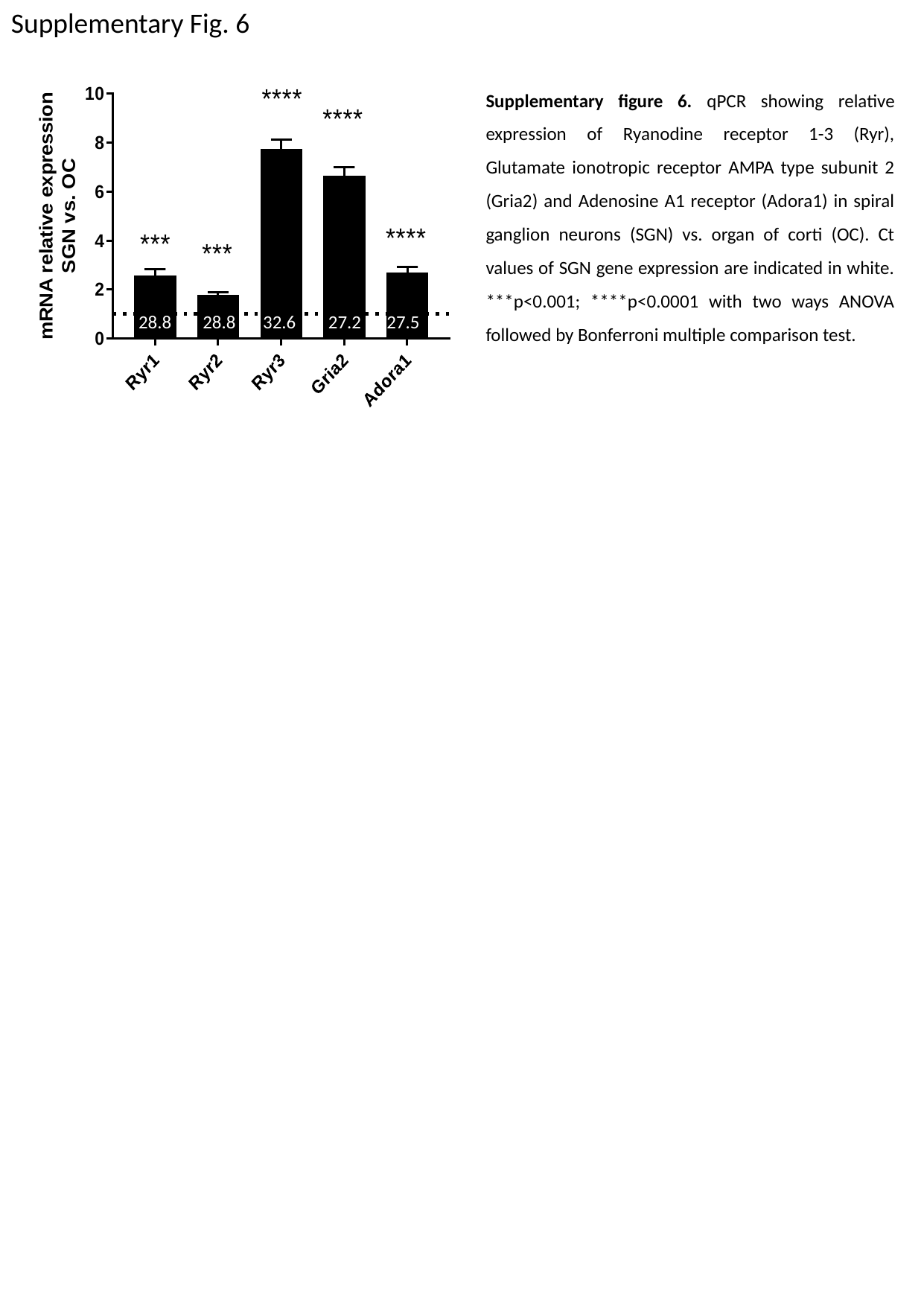

Supplementary Fig. 6
28.8
28.8
32.6
27.2
27.5
Supplementary figure 6. qPCR showing relative expression of Ryanodine receptor 1-3 (Ryr), Glutamate ionotropic receptor AMPA type subunit 2 (Gria2) and Adenosine A1 receptor (Adora1) in spiral ganglion neurons (SGN) vs. organ of corti (OC). Ct values of SGN gene expression are indicated in white. ***p<0.001; ****p<0.0001 with two ways ANOVA followed by Bonferroni multiple comparison test.

## Slide 7
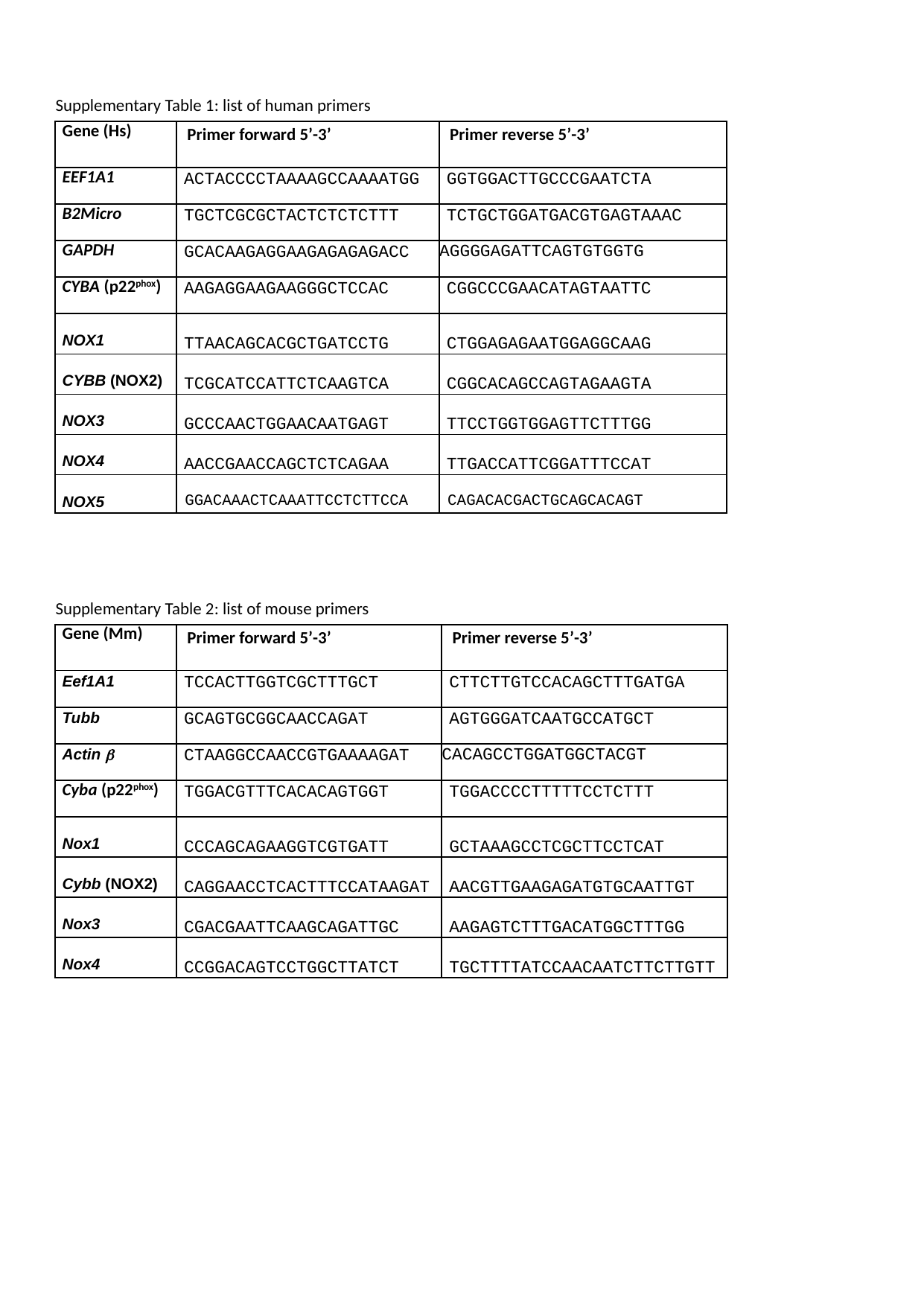

Supplementary Table 1: list of human primers
| Gene (Hs) | Primer forward 5’-3’ | Primer reverse 5’-3’ |
| --- | --- | --- |
| EEF1A1 | ACTACCCCTAAAAGCCAAAATGG | GGTGGACTTGCCCGAATCTA |
| B2Micro | TGCTCGCGCTACTCTCTCTTT | TCTGCTGGATGACGTGAGTAAAC |
| GAPDH | gcacaagaggaagagagagacc | aggggagattcagtgtggtg |
| CYBA (p22phox) | AAGAGGAAGAAGGGCTCCAC | CGGCCCGAACATAGTAATTC |
| NOX1 | ttaacagcacgctgatcctg | Ctggagagaatggaggcaag |
| CYBB (NOX2) | TCGCATCCATTCTCAAGTCA | CGGCACAGCCAGTAGAAGTA |
| NOX3 | Gcccaactggaacaatgagt | Ttcctggtggagttctttgg |
| NOX4 | AACCGAACCAGCTCTCAGAA | TTGACCATTCGGATTTCCAT |
| NOX5 | GGACAAACTCAAATTCCTCTTCCA | CAGACACGACTGCAGCACAGT |
Supplementary Table 2: list of mouse primers
| Gene (Mm) | Primer forward 5’-3’ | Primer reverse 5’-3’ |
| --- | --- | --- |
| Eef1A1 | TCCACTTGGTCGCTTTGCT | CTTCTTGTCCACAGCTTTGATGA |
| Tubb | GCAGTGCGGCAACCAGAT | AGTGGGATCAATGCCATGCT |
| Actin b | CTAAGGCCAACCGTGAAAAGAT | CACAGCCTGGATGGCTACGT |
| Cyba (p22phox) | TGGACGTTTCACACAGTGGT | TGGACCCCTTTTTCCTCTTT |
| Nox1 | CCCAGCAGAAGGTCGTGATT | GCTAAAGCCTCGCTTCCTCAT |
| Cybb (NOX2) | CAGGAACCTCACTTTCCATAAGAT | AACGTTGAAGAGATGTGCAATTGT |
| Nox3 | CGACGAATTCAAGCAGATTGC | AAGAGTCTTTGACATGGCTTTGG |
| Nox4 | CCGGACAGTCCTGGCTTATCT | TGCTTTTATCCAACAATCTTCTTGTT |
